# Dominant Role of Nucleotide Substitution in the Diversification of Serotype 3 Pneumococci over Decades and during a Single Infection

**DOI:** 10.1371/journal.pgen.1003868

**Published:** 2013-10-10

**Authors:** Nicholas J. Croucher, Andrea M. Mitchell, Katherine A. Gould, Donald Inverarity, Lars Barquist, Theresa Feltwell, Maria C. Fookes, Simon R. Harris, Janina Dordel, Susannah J. Salter, Sarah Browall, Helena Zemlickova, Julian Parkhill, Staffan Normark, Birgitta Henriques-Normark, Jason Hinds, Tim J. Mitchell, Stephen D. Bentley

**Affiliations:** 1 Pathogen Genomics, The Wellcome Trust Sanger Institute, Wellcome Trust Genome Campus, Hinxton, Cambridge, United Kingdom; 2 Center for Communicable Disease Dynamics, Department of Epidemiology, Harvard School of Public Health, Boston, Massachusetts, United States of America; 3 Institute of Microbiology and Infection and School of Immunity and Infection, College of Medical and Dental Sciences, University of Birmingham, Birmingham, United Kingdom; 4 Bacterial Microarray Group, Division of Clinical Sciences, St. George's Hospital, University of London, London, United Kingdom; 5 Institute of Infection, Immunity and Inflammation, College of Medical, Veterinary and Life Sciences, University of Glasgow, Glasgow, United Kingdom; 6 Department of Microbiology, Tumour and Cell Biology, Karolinska Institutet, Stockholm, Sweden; 7 Dept. of Laboratory Medicine, Division of Clinical Microbiology, Karolinska University Hospital, Stockholm, Sweden; 8 National Institute of Public Health, Prague, Czech Republic; 9 Department of Medicine, University of Cambridge, Addenbrooke's Hospital, Cambridge, United Kingdom; Inserm U722, France

## Abstract

*Streptococcus pneumoniae* of serotype 3 possess a mucoid capsule and cause disease associated with high mortality rates relative to other pneumococci. Phylogenetic analysis of a complete reference genome and 81 draft sequences from clonal complex 180, the predominant serotype 3 clone in much of the world, found most sampled isolates belonged to a clade affected by few diversifying recombinations. However, other isolates indicate significant genetic variation has accumulated over the clonal complex's entire history. Two closely related genomes, one from the blood and another from the cerebrospinal fluid, were obtained from a patient with meningitis. The pair differed in their behaviour in a mouse model of disease and in their susceptibility to antimicrobials, with at least some of these changes attributable to a mutation that up-regulated the *patAB* efflux pump. This indicates clinically important phenotypic variation can accumulate rapidly through small alterations to the genotype.

## Author Summary

*Streptococcus pneumoniae* (‘the pneumococcus’) is a bacterium commonly found asymptomatically in the human nasopharynx that represents a common cause of diseases such as pneumonia, bacteraemia and meningitis. Some strains have been found to exchange DNA with other bacteria at a high rate. However, serotype 3 pneumococci are unusual both in not exhibiting much genetic variation and causing disease with a comparatively high relative rate of mortality. Here we used whole genome sequencing to characterise 82 serotype 3 pneumococci, finding that the majority of the population accumulate variation very slowly. However, comparing two isolates from a single case of disease revealed a small number of mutations had occurred over a short period of time. These resulted in differences in the activity of several genes, including two encoding a drug efflux pump. The pair of isolates was found to differ in their tolerance of different antimicrobial compounds and their behaviour in a mouse model of disease. However, moving the mutation that caused the change in resistance into a distantly-related pneumococcus failed to fully replicate the other changes in behaviour, which indicates that interpretation of the impact of mutations in different strains of diverse bacterial species will be difficult.

## Introduction

*Streptococcus pneumoniae* is a human nasopharyngeal commensal and respiratory pathogen responsible for a high burden of morbidity and mortality worldwide. Serotype 3 was one of the earliest pneumococcal capsule types to be identified [1]. For some time, there was dispute over whether these bacteria should be considered a separate species, named *Streptococcus* or *Pneumococcus mucosus* [2], and whilst such a separation cannot be justified on the basis of genetic divergence it does have distinctive morphological and epidemiological traits. Bacterial colonies of this serotype have a characteristic mucoid phenotype when grown on agar, as the cellobiuronic acid polymeric chains of the capsule are not covalently attached to the cell wall [3], [4]. Unusually for *S. pneumoniae*, the risk of serotype 3 disease increases with age [5]–[8], which may relate to the high immunogenicity of the capsule antigen in young children [9].

Importantly, disease caused by this serotype has been consistently associated with a high relative risk of mortality in humans [5], [6], [10]–[13], and correspondingly strains of this serotype are amongst the quickest to cause death in a mouse model of bacteraemia [14]. The high level of mortality may stem from the high frequency with which serotype 3 isolates cause extrapulmonary manifestations of pneumococcal infection [15], with evidence that the serotype is associated with causing brain abscesses [16]–[18]. Whether these characteristics are the consequence of the capsule or the genetic background itself is difficult to study, because few genotypes are stably associated with the type 3 capsule.

The most common of these in the multilocus sequence type database [19] is represented by isolates of, or closely related to, sequence type 180 (ST180); this lineage is therefore termed clonal complex 180 (CC180), or the Netherlands 3–31 (PMEN31) clone [20]. This lineage is geographically highly widespread, having been found across Europe [21], Japan [22] and North and South America [21], [23]–[25]. Although it is not associated with penicillin resistance, macrolide resistant representatives of the genotype have been identified [22]. Since 2001, this lineage has been observed to increase in prevalence among invasive disease isolates from the USA following the introduction of the heptavalent conjugate vaccine, which does not protect against serotype 3 pneumococci [23]. Higher valency anti-pneumococcal conjugate polysaccharide vaccine formulations targeting this capsule, such as the recently introduced 13-valent vaccine, appear to trigger only weak immune reactions to their serotype 3 components, hence it remains unclear how effective they will be against such pneumococci [26], [27]. Therefore to better characterise this unusual and important lineage, a complete reference genome was generated and compared to sequence data from 81 other representatives.

## Results

### Whole genome phylogeny of clonal complex 180

The complete genome of *S. pneumoniae* OXC141, a serotype 3 ST180 carriage isolate from a child in Oxford, was generated using a combination of 454 and capillary sequence data. The chromosome was found to be 2,036,867 bp long and contained 1,986 coding sequences (CDSs; including 153 pseudogenes), alongside many small interspersed repeat elements: 122 BOX elements, 106 RUP elements and 29 SPRITE repeats [28]. Two putative mobile genetic elements could be identified: the 34 kb prophage φOXC141 [29] and a 6.3 kb island likely to be related to, or derived from, an integrative and conjugative element (ICE) [30]. Two further large, distinctive gene clusters were also evident: a ∼22 kb region directly upstream of *pspA* appearing to encode multiple bacteriocin production systems, and the variable region of Pneumococcal Pathogenicity Island 1 (PPI-1) containing a ∼25 kb long set of miscellaneous metabolic genes [31].

In order to ascertain the level of variation in gene content across CC180, comparative genomic hybridisation was used to select six further representatives to be sequenced using a combination of 454 and capillary technologies. These were complemented by an international sample of 75 isolates from Europe and North America sequenced as multiplexed libraries using the Illumina GAII platform (Table S1). A phylogenetic analysis of this collection was performed as described previously [32].

**Figure 1:**
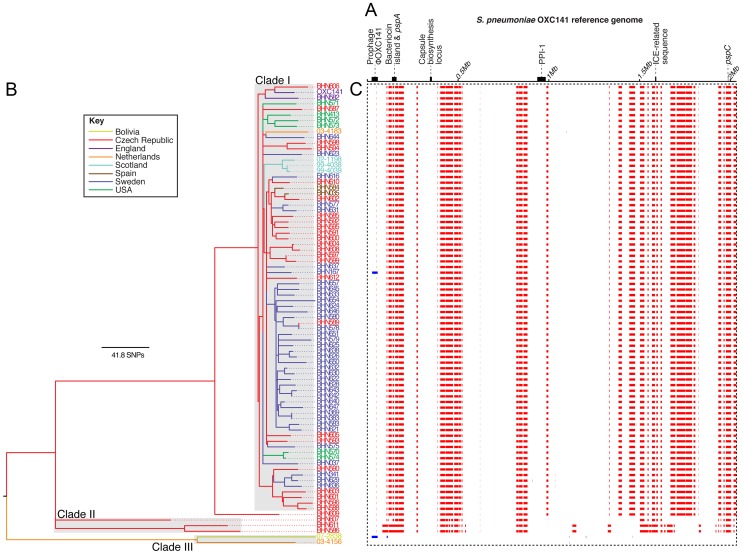
Phylogenetic analysis of the clonal complex 180 isolates. (A) A simplified representation of the 2,036,867 bp genome of *S. pneumoniae* OXC141 with tick marks every 0.5 Mb. The loci encoding the proteinaceous antigens PspA and PspC, as well as the capsule biosynthesis locus, are labelled, as are the prophage φOXC141, a putative ICE-related sequence, the ∼22 kb bacteriocin island and Pneumococcal Pathogenicity Island 1 (PPI-1). (B) A maximum likelihood phylogeny of the serotype 3 isolates based on the vertically inherited substitutions not introduced through recombination events. The tree and taxa are coloured according to their geographical location. (C) The panel surrounded by the dotted line contains one row for each isolate in the tree, with a column for each base in the reference genome. Red blocks indicate recombination events reconstructed as occurring on an internal branch, hence are shared by more than one isolate through common descent, while blue blocks indicate recombinations unique to one particular isolate.

A total of 12,605 substitutions were reconstructed as occurring over the history of the lineage, of which 77% were introduced by 82 recombinations (two acquisitions of prophage, one recombination affecting the ICE-related sequence and 79 putative homologous recombinations; Figure 1). The lengths of the homologous recombinations were exponentially distributed with a mean length of 11.5 kb (Figure S1), each introducing a mean of 116 substitutions. However, a highly irregular pattern of sequence imports is clear across the phylogeny, with the majority of the variation arising on a small number of long branches separating three clades (labelled I, II and III in Figure 1). Single nucleotide polymorphisms (SNPs) were detected at just 1,925 sites in clade I, which contains all but six isolates. Only one prophage integration, and eleven putative homologous recombinations of a mean length of 20.4 bp, are detected in this clade, resulting in an overall per site *r/m* (the ratio of substitutions accumulating through recombination relative to those occurring through point mutation) of 0.07, approximately two orders of magnitude below the equivalent value of 7.2 calculated for the PMEN1 lineage using the same method [32]. This absence of any signs of extensive sequence import into clade I through homologous recombination was confirmed by analysing the whole genome alignment with BRATnextgen (Figure S2) [33].

The tree structure suggests clade I is an expansion emerging from a more diverse background of isolates, although the geographic bias of the sample makes it difficult to draw general demographic conclusions. Hence the apparent absence of widespread recombination may reflect a short evolutionary history of clade I, in which there has been little time to horizontally acquire novel sequence. However, a coalescent analysis of the phylogeny indicated this clade is likely to have last shared a common ancestor in about 1947 (95% credibility interval: 1907–1970). The same analysis predicted an overall age of around 330 years (95% credibility interval: 592-177 years) for CC180. The implied mean substitution rate of 3.65×10^−7^ substitutions per site per year (95% credibility interval 1.77×10^−7^–5.58×10^−7^ substitutions per site per year) is slower than that of PMEN1 (1.57×10^−6^ substitutions per site per year), which may be the consequence of purifying selection having more time to remove deleterious substitutions in this older lineage [34]. Nevertheless, it appears clade I is of at least an equivalent age, and very likely older, than the multidrug-resistant lineages in which extensive horizontal sequence transfer has been observed, leading to the impression that the genotype has been effectively ‘frozen’ over decades.

### Extensive variation in the accessory genome

**Figure 2:**
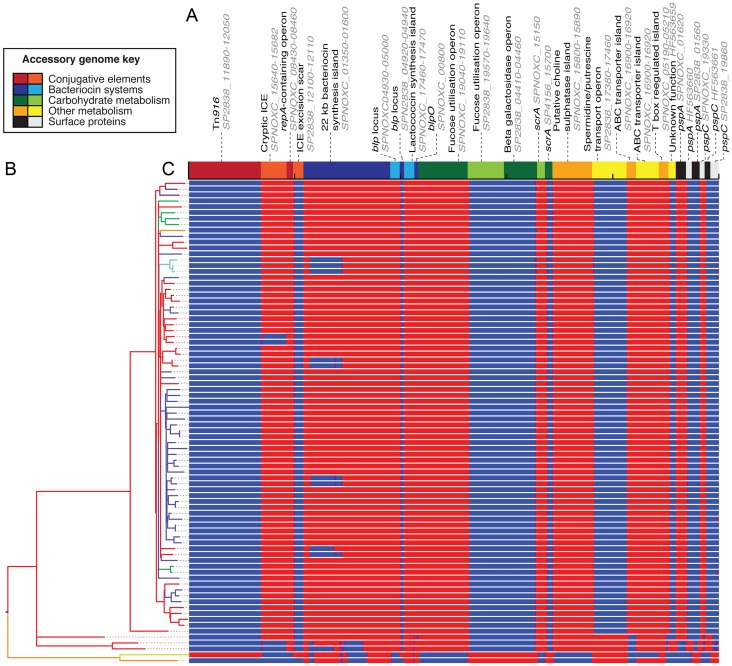
Distribution of accessory genome loci. (A) The non-prophage accessory genome loci, identified through comparison of genome assemblies, are displayed as blocks of alternating colours. The loci are coloured and grouped according to their functional annotation. All sequences are labelled with their position in a publicly available genome, or their ENA accession code; some have been trimmed to remove insertion sequences, to avoid read mapping artefacts. (B) The maximum likelihood phylogeny, as displayed in Figure 1. (C) This panel is composed of one row per taxon, each representing the output of mapping the sequence reads from an individual sample against the accessory genome loci. This is displayed as a heatmap that is blue where there is no read mapping, indicating an absence of a locus, changing to red where there is read mapping indicating the locus is present (scaled to a maximum of 100 reads per million reads mapped).

Identification of variable loci through comparison of *de novo* assemblies revealed extensive overall variation contrasting with stability within clade I, in agreement with the phylogeny (Figure 2). For instance, the widespread presence of prophage **φ**OXC141 indicates it was acquired by an ancestor of clade I and subsequently deleted on at least seven independent occasions based on this sample (Figure S3). This virus, observed to form intact virions following induction with mitomycin C [29], appears to be active during *in vitro* culture based on the sequence read coverage compared to the rest of the genome (Figure S4). One of the few genotypes to have lost **φ**OXC141, BHN167, is the only isolate in clade I showing evidence of having acquired a novel prophage (**φ**BHN167), which is highly divergent from the others in the collection. Nevertheless, the level of flux of such elements appears much slower than observed in PMEN1 [32].

Also stable within clade I are the proteinaceous antigens PspA and PspC (Figure 2), which are highly variable across the species [35], [36]; however, divergent alleles are evident in the other CC180 clades. Similarly, loci encoding putative bacteriocin synthesis gene clusters differ between the annotated clades, with the large island upstream of *pspA* varying even within clade I: one deletion of approximately 10 kb, seemingly driven by a recombination between the very similar regulatory genes SPNOXC01360 and SPNOXC01480, is homoplasic within the sample. Metabolic operons, by contrast, only exhibited considerable between-clade variation. This suggests accessory loci are gained over the long periods of divergence separating clades, with subsequent occasional deletion observed over shorter timescales, although only clade I is sampled with sufficient density to study this pattern in detail.

Just one instance of antibiotic gene acquisition is evident in the collection. This is an insertion of a Tn*916*-type element [37], carrying the *tetM* tetracycline resistance gene, into the Bolivian strain *S. pneumoniae* 07-2838. Furthermore, no fluoroquinolone resistance polymorphisms within the topoisomerase genes *gyrA*, *gyrB*, *parC* and *parE* could be found, despite being homoplasic in the phylogenies of such disparate genotypes as *S. pneumoniae* PMEN1 [32], *Staphylococcus aureus* ST239 [38] and *Salmonella* Typhi [39]. These can arise spontaneously through point mutation, and therefore seemed likely to be observed even in cases where recombination is not common.

### Within-patient variation of pneumococci

However, there is one SNP in the phylogeny associated with antibiotic resistance: a polymorphism 46 bp upstream of the start codon of the genes encoding the ABC-type efflux pump PatAB [40] distinguishing the closely-related clade I isolates *S. pneumoniae* 99-4038 and 99-4039. These were cultured from a single patient with meningitis: 4038 was taken from the bloodstream, and 4039 from the cerebrospinal fluid (CSF). They were selected for sequencing from a screen of isolate pairs, each obtained from the same patient, owing to them exhibiting the most pronounced difference in transcriptional profiles. Improved high quality draft genomes of both isolates revealed a small number of polymorphisms largely concentrated in repetitive or hypervariable regions of the chromosome that likely represent difficulties in assembly or mutation during *in vitro* culture (Table S2). Nevertheless, three high-confidence mutations can be identified as distinguishing the pair in addition to the base substitution upstream of *patAB*: a synonymous change in the putative regulatory protein SP4038_08190, a non-synonymous L227M substitution in the putative hydrolase SP4038_11450, and a frameshift mutation truncating the putative phosphohydrolase SP4038_15170 in 4038.

**Figure 3:**
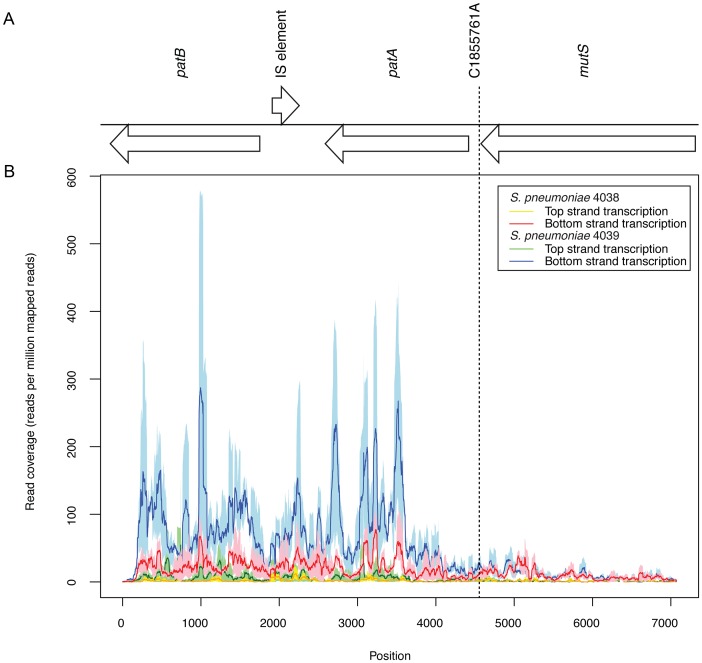
Expression of the *patAB* locus. (A) Annotation of the *patAB* gene cluster, with the intervening IS element remnant on the opposite strand, and the upstream *mutS* gene. The position of the C1855761A polymorphism distinguishing *S. pneumoniae* 4038 and 4039 is indicated. (B) Graph showing Illumina read coverage, standardised as a plot of reads mapping per million reads mapped, from RNA-seq experiments. Each line shows the mean value from three replicates surrounded by a shaded area representing the minimum and maximum values. The red and orange lines represent transcription of the two strands of the genome in 4038, and the blue and green lines represent the transcription of the two strands of the genome in 4039.

The first test of whether these two isolates from distinct anatomical sites differed in their transcriptional profiles had involved hybridising RNA samples extracted from 4038 and 4039 during *in vitro* growth to a microarray based on the genomes of *S. pneumoniae* TIGR4 and R6. This revealed the significant differences in their patterns of gene expression detailed in Table S3. The *patAB* genes were found to be approximately five-fold upregulated in 4039, while the RNA polymerase gene *rpoE* and translation machinery genes *rplS*, *rpsB* and *tsf* were each expressed at a lower level. RNA sequencing (RNA-seq) of three further independent paired samples from the two isolates grown to an OD_600_ of 0.6 in Brain-Heart infusion were then used to characterise these differences more precisely. This experiment confirmed the results for the previously mentioned genes, with *patAB* this time exhibiting approximately four-fold greater transcription in 4039 (Table S4). The change in expression appeared to coincide with the SNP distinguishing the isolates (the allele of this locus found in 4039 is henceforth referred to as the *patAB* upstream SNP, PUS; Figure 3), with both genes co-transcribed as an operon despite the intervening degenerate transposase sequence being encoded on the complementary strand of the genome.

Overall, RNA-seq found 54 CDSs differed significantly in their level of expression between the two isolates, grouped into 11 gene clusters and 18 singleton CDSs based on the genome sequences. The chaperone genes *dnaK*, *grpE* and *clpL* were found to be transcribed at a lower level in 4039. By contrast, many genes involved in nucleotide biosynthesis and acquisition exhibited higher levels of expression in 4039. These include many genes in the *pur* operon, the adenylate kinase *adk*, the guanine monophosphate synthase *guaA* and the putative xanthine or uracil transporter SP4038_03000.

### Patterns of gene expression

The RNA-seq data also provided information on the expression of some of the distinctive genetic loci associated with CC180 genome sequences. Congruent with the DNA sequence coverage mapping, active transcription of the **φ**OXC141 prophage's lytic cycle genes was observed (Figure S4), although transcription of the lysogeny module was also apparent, indicating a mixed population with active phage replication in some cells. Overall, there was no evidence of discrete states of transcription (Figure S5), consistent with studies in other species [41]. When categorised according to gene function, it is clear the most highly expressed genes, in a sense direction, are those involved in glycolysis, central metabolism, transcription and translation (Figure S6). By contrast, those CDSs associated with the highest levels of antisense transcription relative to sense activity were pseudogenes (Figure S7), with a significantly higher proportion of these gene fragments transcribed in a predominately antisense direction in comparison to intact CDSs (Fisher's exact test, *p* value = 6.14×10^−11^). This indicates a level of decay of transcriptional regulation of such loci in the chromosome, with the degenerate IS element in the *patAB* operon a clear example (Figure 3).

There are also two notable examples of functional operons being highly expressed in an antisense manner. One is a gene cluster encoding a series of restriction endonuclease system genes (SP4038_07830-07890). Another is the *comCDE* operon, encoding the competence stimulating peptide precursor and its cognate receptor. Antisense transcription of these genes, crucial for activating the competence system for DNA uptake, contrasts with their predominately sense transcription in a sample extracted from the PMEN1 isolate *S. pneumoniae* ATCC 700669 under similar conditions [42]. Such regulation may indicate one reason that so few transformation events are observed in clade I of CC180. Few other reasons are obvious from the genome sequences alone, with the major elements of the competence machinery appearing intact in all isolates with the exception of the isolates BHN644, BHN587 and BHN605, with premature stop codons detected in the *comD* sensor kinase, *comFA* helicase and *comEA* transport system, respectively.

### Variation in resistance and virulence

The PUS is the polymorphism likely to be making the biggest contribution to the observed changes in transcription, as it is closely linked to the genes undergoing the greatest change in expression, *patAB*. This locus has previously found to vary in activity between clinical isolates [43] both in the presence and absence of inducing chemicals such as fluoroquinolones and mitomycin C [44]. Therefore it seems likely these genes are likely to be subject to altered selective pressures during the progression of disease. The impact of the other polymorphisms differentiating 4038 and 4039 is more difficult to understand. The substitution in the putative regulatory gene is synonymous, and therefore unlikely to contribute to alterations in expression. Ascertaining the effect of the polymorphisms in phosphohydrolases is difficult given the unknown impact of the changes on gene products with functions that have yet to be thoroughly characterised.

Isolates with elevated levels of *patAB* expression are observed to have reduced susceptibility to fluoroquinolones and other antimicrobials, including linezolid [40], reserpine [45], acriflavine, berberine, ethidium bromide [46] and the dye Hoescht 33352 [43]. The sensitivity of isolates 4038 and 4039 to a wide range of antimicrobial compounds and osmolytes at a range of concentrations was therefore tested using phenotype microarrays [47]. A significant difference between the isolates could be detected in 25 cases; all resulted from *S. pneumoniae* 4039 exhibiting greater resistance to antimicrobial agents (Table S5), with no evidence that increased expression of *patAB* affected the bacterium's membrane integrity based on the sensitivity to concentration gradients of osmolytes. Isolate 4039 appeared to exhibit higher levels of tolerance of the toxic anions boric acid, sodium metaborate and sodium bromide, which could indicate elevated efflux pump activity. Other antimicrobials that resulted in 4039 having a significantly elevated respiratory rate relative to 4038 were, like fluoroquinolones, nucleic acid intercalators: proflavine and the related compound acriflavine, along with 5,7-dichloro-8-hydroxyquinoline and 6-mercaptopurine, for which a significant difference was observed at three of the four tested concentrations. Some of the other compounds identified as distinguishing the isolates, such as pentachlorophenol, crystal violet and 2,4-dinitrophenol, are known to act as uncouplers of proton gradients. This could be the consequence of such compounds being removed from the cell by the PatAB efflux pump or, as PatAB is an ABC transporter and therefore not dependent on electrochemical gradients as some other pumps are, this difference in respiration rate may represent a more general change in the overall pattern of molecule efflux. In conjunction with the previously reported elevated resistance to a variety of compounds, these data indicate elevated PatAB activity is likely to increase tolerance to a broad spectrum of antimicrobials.

**Figure 4:**
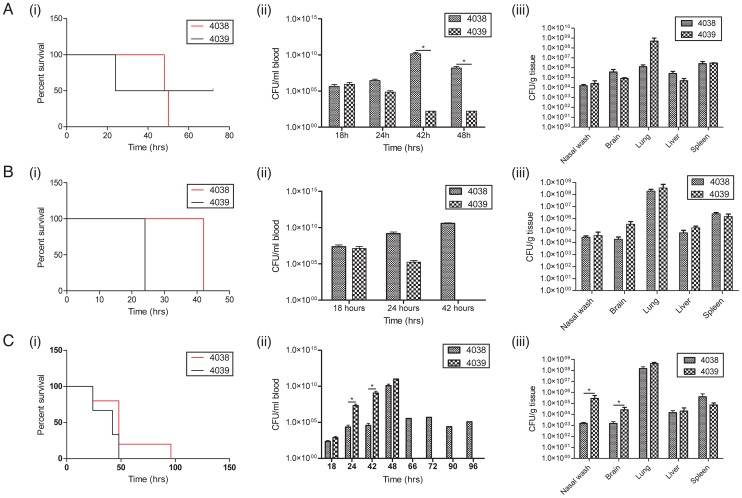
Differences in virulence of *S. pneumoniae* 4038 and 4039, clinical isolates in which the latter contains a SNP upregulating *patAB*. The results of three experiments are shown: (A) mice inoculated with 10^2^ CFU intraperitoneally (n = 5), (B) mice inoculated with 10^4^ CFU intraperitoneally (n = 5) and (C) mice challenged with 10^6^ CFU intranasally (n = 5). For each, the displayed outcomes are: (i) survival curves, (ii) level of bacteraemia and (iii) counts of the two isolates in different organs. Significant differences between viable cell counts, calculated at a *p*<0.05 level using a T test, are indicated by asterisks.

To test whether these phenotypic alterations impacted on the virulence of the two isolates, both were assayed in the mouse model of invasive pneumococcal disease (Figure 4). When 10^2^ colony forming units (CFU) were introduced through intraperitoneal inoculation there was no significant difference in the survival time in animals infected with either isolate. However, animals infected with 4038 had 10^10^ CFU/mL in the blood at the time of death whereas animals infected with 4039 had very low bacterial counts in the bloodstream. When the intraperitoneal challenge dose was increased to 10^4^ CFU all animals infected with 4039 reached the end point by 24 h, whereas those infected with 4038 lasted until 42 h (Wilcoxon test, n = 4, *p* = 0.0082), although in this experiment there were no significant differences in the organ distribution of the isolates. When a model of pneumonia was induced by intranasally inoculating animals with 10^6^ CFU there was no difference in survival time in animals infected with either isolate. However mice infected with strain 4039 developed higher levels of bacteraemia earlier in the infection with significantly higher levels in the blood after 24 and 48 h post infection; 4039 was also present in significantly higher numbers in the nasopharynx and brain relative to 4038. Hence the observed significant differences in tissue distribution of each genotype were heavily dependent upon the route of inoculation.

### Complex consequences of *patAB* regulatory changes

**Figure 5:**
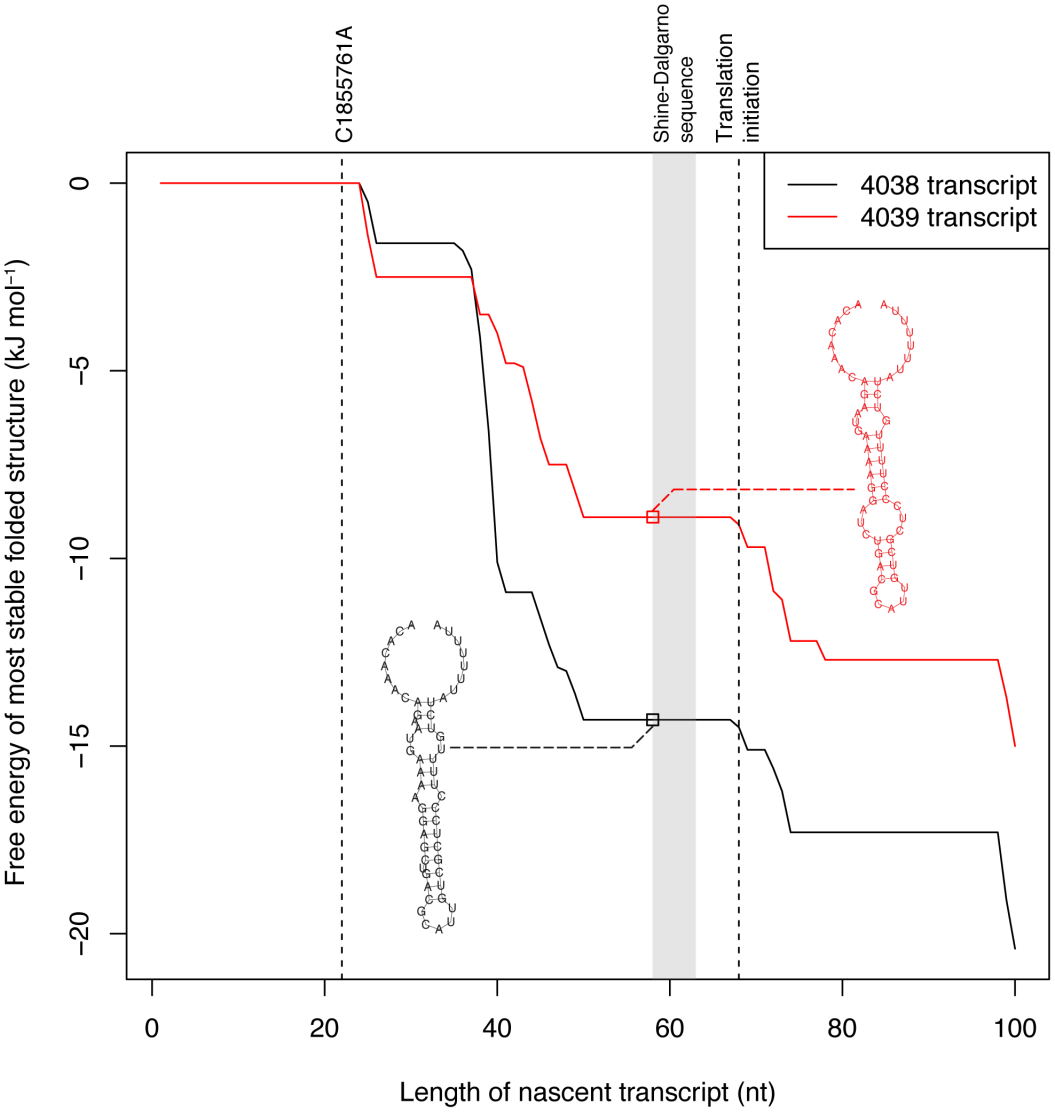
Leader sequence upstream of *patAB*. The graph represents the free energy of the most stable folded form of the 5′ region of the *patAB* transcript, as it extends from the promoter, in 4038 (black line) and 4039 (red line). The positions of the C1855761A polymorphism (PUS) distinguishing the two isolates and translation initiation site are indicated by the dashed lines, while the Shine-Dalgarno sequence is represented by the shaded region. The terminator-like hairpin structures formed by both transcripts as the RNA reaches the point at which the Shine-Dalgarno sequence is transcribed are displayed; it is evident that the mutation in 4039 is predicted to greatly weaken the hairpin loop of this secondary structure.

Analysis of the region upstream of the *patAB* operon revealed promoter motifs close to the consensus sequences appearing to initiate transcription 67 nt upstream of the *patA* start codon. The consequent 5′ untranslated region is predicted to fold into a bulged hairpin loop followed by a run of uridine residues, indicating it could function as a terminator (Figure 5). This suggests a simple transcriptional attenuation regulatory mechanism: any signal that destabilizes this hairpin seems likely to increase the transcription of the downstream CDSs. The transcription of these genes is known to be increased by compounds that can intercalate nucleic acids, which have been found to induce conformational changes in bulged RNA hairpin loops [48]. Whether regulation is via such a direct interaction, or involves other factors, it would seem appropriate that *patAB* would be induced through a relatively non-specific signal given its apparent ability to remove a range of intercalating compounds from the cell.

The PUS distinguishing 4038 and 4039 is predicted to destabilize this hairpin loop (Figure 5), likely reducing any transcriptional attenuation and providing a potential explanation for the observed difference in expression levels. To study the consequences of this SNP in isolation, the genetically tractable *S. pneumoniae* TIGR4 strain [49], which has the same sequence upstream of *patAB* as 4038, was transformed with the region upstream of *patA* from isolates 4038 and 4039. Isolation of fluoroquinolone resistant colonies that had acquired the PUS from 4039 (Figure S8), followed by characterisation with E tests, indicated the PUS increased the ciprofloxacin MIC from 1 mg L^−1^ to 4 mg L^−1^, while the MICs of 4038 and 4039 were 0.75 mg L^−1^ and 2 mg L^−1^, respectively (Table S6). Hence the PUS elevates resistance in both backgrounds, with the MIC also being determined by the rest of the isolate's genotype.

Comparing the expression profile of *S. pneumoniae* TIGR4 and TIGR4^PUS^ using a microarray revealed the *patAB* operon was upregulated by a relatively small degree, (∼2.5 fold) with no evidence of other significant differences, such as those observed between 4038 and 4039 (Table S7). Furthermore, comparison of TIGR4 and TIGR4^PUS^ using the mouse model of disease found no significant difference in the rate at which the mice reached the end point of infection, nor the final tissue distribution of bacteria (Figure S9).

## Discussion

The patterns of evolution observed across this collection of CC180 isolates can be seen as reflecting the impact of selection on accumulated variation over different timescales. Polymorphisms are acquired horizontally at a heterogeneous rate across the tree, with deep branching clades distinguished by extensive sequence diversity arising through point mutation, transformation and the movement of mobile genetic elements. Within clade I, however, horizontal sequence transfer makes almost no contribution to the evolution of the genotype, which is ‘frozen’ in a stable form. Whether this sample, with its geographical bias towards Europe, reflects the overall population structure of CC180 is unclear; denser sampling of clades II and III samples may indicate the recombinations occurred gradually in these lineages, with clade I being atypically stable. Nevertheless, as the number of point mutations in clade I indicates it has been diverging over decades, it remains clear that transformation events are accumulating at a very low rate in this lineage.

Contrasting with this slow overall net rate of diversification, four high-confidence polymorphisms could be identified distinguishing the isolates 4038 and 4039 from a single patient. These mutations (the PUS, a synonymous change in a DNA-binding protein, a non-synonymous substitution in one putative hydrolase and a truncation of another) may well represent normal neutral diversification that is purged by purifying selection, or lost by drift, over longer timescales. Alternatively, they may be the product of adaption to the change in environment encountered during the course of invasive pneumococcal disease. The latter scenario seems most likely in the case of the PUS, based on the diversity in *patAB* expression observed in surveys of clinical isolates, and a previous observation of resistance emerging during serotype 3 pneumococcal disease (although the underlying mechanism of resistance, and genetic background of the strain, are not known [50]). However, only with more detailed characterisation of within-host evolution in different anatomical niches will be possible to systematically answer this question. Such studies are beginning to be performed: for instance, mutations have been observed to accumulate over short timescales during carriage and disease caused by *Staphylococcus aureus* [51], [52], and *Escherichia coli* [53], [54], with some of the observed changes associated with differences in mouse models of virulence.

The alternative explanation to adaptation, that these SNPs represent neutral mutation that does not persist over longer timescales, would suggest that purifying selection might contribute to maintaining the antibiotic sensitive phenotype. However, lack of resistance to other antibiotics may also be attributable to the inability to acquire the requisite sequences. For instance, the Tn*916*-type tetracycline resistance element observed in one isolate indicates it is possible for the genotype to acquire ICEs; the rarity of this resistance in CC180 may just represent such transfers being infrequent, or it may be that selection is the more important factor in eliminating isolates with such transposons. Perhaps the most interesting case is the absence of any large recombination events in clade I having precluded the development of β-lactam resistance through modification of penicillin binding proteins. Given the hypothesised ‘purging’ of point mutations through selection suggested by the relative rates of base substitution during disease and over the phylogeny, the ‘frozen’ genotype could be the consequence of recombination events being similarly removed by selection in CC180. Alternatively, it may simply be that CC180 imports DNA at a low rate relative to other pneumococci, resulting in the stalling of clade I's diversification. Reasons behind CC180's lack of transformation could be the consequence of inhibition by the mucoid capsule; this has been observed to slow, but not entirely inhibit, transformation in non-CC180 serotype 3 pneumococci [55]. Another physiological explanation for a slowed rate of DNA uptake could be the antisense transcription of the competence-inducing *comCDE* genes, if this were also observed to occur *in vivo*. Alternatively, the explanation could reflect the lineage's epidemiology. Serotype 3's increased prevalence in adults (inferred from disease frequency; *e.g.* [5]) may mean it has relatively little opportunity to import sequence diversity owing to the reduced chance of co-colonising with most other genotypes, which are found more frequently in children.

Nevertheless, diversification through point mutations alone can still result in large phenotypic differences, as demonstrated by the behaviour of isolates 4038 and 4039 in the mouse model of disease. Following intraperitoneal inoculation, 4038 caused high-level bacteraemia, whereas 4039 was cleared from the bloodstream; this is interesting given the isolation of 4038 from the blood of the patient. By contrast, 4039 rises faster in the blood following intranasal inoculation, and is found at higher levels in the brain, perhaps suggesting an enhanced ability to traverse anatomical barriers could explain its presence in the CSF of the original patient. Hence polymorphisms distinguishing the pair may represent adaptation to different anatomical niches over a short timescale.

One potential explanation of this multiplicity of phenotypic differences between 4038 and 4039 is that they all represent consequences of the PUS. This would suggest the PUS is likely to be atypical in having such a strong impact on phenotype, owing to its effects on the regulation of a broad-spectrum pump induced by a wide range of compounds. However, such a hypothesis must also account for the failure of the SNP to cause the same changes in the TIGR4 strain, perhaps owing to genetic interactions with other loci modulate the impact of the mutation. The baseline differences in fluoroquinolone susceptibility between TIGR4 and 4038 provide some evidence for this. Hence further work investigating the activity of the PatAB pump in different backgrounds seems likely to be informative. Recent characterisation of a *patAB* knockout mutant of the *S. pneumoniae* R6 strain using a phenotype microarray found the mutant to have increased susceptibility to fluoroquinolones and acriflavine, consistent with the phenotype microarray work in this study, as well as tetracyclines, consistent with the results of the Vitek 2 analysis (Table S6). Although overlapping, the results were not entirely consistent [56], which could represent the consequences of the contrasting genetic manipulations of overexpression versus removal of the encoding genes, differences in analytic approaches, or the influence of genetic background upon the consequences of a polymorphism.

Combining the data in this study suggests that the PUS has an unusually strong impact on phenotype in the CC180 background in particular. It is notable that several of the transcriptional changes observed in CC180, but not TIGR4, imply the PUS causes dysregulation of purine metabolism. This could be a consequence of metabolites involved in this pathway being removed from the cell by the pump. Evidence for this hypothesis is provided by the differences in respiration between 4038 and 4039 in the presence of the antimicrobial close purine analogue 6-mercaptopurine. One putative reason why the CC180 genotype might be unusually sensitive to such a perturbation of this aspect of metabolism is that expression of the lysogeny module of φOXC141 drives antisense transcription of the adjacent *purA* gene, crucial for purine generation, in 4038 and 4039 (Figure S4).

Alternatively, one or more of the other polymorphisms distinguishing 4038 and 4039 may cause the additional effects the phenotypically differentiate this pair, but not TIGR4 and TIGR4^PUS^. While the synonymous change in a regulatory protein seems unlikely to have a large effect on measureable traits, the observation of two mutations in phosphohydrolases could potentially indicate selection for a particular alteration in cellular biochemistry. Further investigation of cases of within-patient evolution will be invaluable in highlighting whether any of these SNPs are commonly identified occurring during the progression of pneumococcal disease. Under such circumstances where more than one of four observed polymorphisms were to measurably affect the bacterial phenotype, it becomes difficult to make the general assumption that the majority of observed polymorphisms in the pneumococcal chromosome are selectively neutral. This could be interpreted as supporting a role for purifying selection in removing a high proportion of point mutations from the genotype, congruent with the relative rates of mutation observed within the patient and over the history of clade I. Ultimately, either explanation – that the background into which the SNP is introduced is important, or a high proportion of SNPs significantly impact on the overall phenotype - indicate, even with whole genome sequences, inferring the phenotypic consequences of even small differences between strains will be a complex task.

## Materials and Methods

### Ethics statement

All animal work was approved by University of Glasgow ethics committee and was conducted under National Guidelines under Home Office Project Licence number 60/3703.

### Genome sequencing

Seven isolates were selected for sequencing from an international collection on the basis of the diversity of their accessory genomes, as assayed through comparative genome hybridisation. The reference genome of *S. pneumoniae* OXC141 was sequenced to completion. For the assembly of the *S. pneumoniae* OXC141 genome, two shotgun libraries using the pSMART vector (insert sizes 4–6 kb and 6–94 kb) and five shotgun libraries using the pUC vector (insert sizes 0.8–1.2 kb, 1.2–2 kb, 1.2–1.6 kb, 2–2.5 kb and 2–4 kb) were sequenced using BigDye terminator chemistry on ABI3730 sequencing machines (Applied Biosciences) Capillary sequence data were assembled using phrap2gap [57], and multiple rounds of PCR used to close gaps and complete the genome to a finished standard [58].

Isolates *S. pneumoniae* 99-4038, 99-4039, 02-1198, 03-4183, 03-4156 and 07-2838 were sequenced to improved high quality draft status [58]. ABI3730 capillary sequence reads from shotgun libraries cloned into pOTWI2 vectors were combined with 454 FLX sequence data to give hybrid assemblies using Newbler (http://my454.com/products/analysis-software/index.asp) and phrap2gap. Multiple rounds of PCR were used to close gaps. For isolates *S. pneumoniae* 99-4038 and 99-4039, 75 nt paired end Illumina sequence data (ENA accession codes: 4038, ERR052247; 4039, ERR052248) from multiplexed libraries sequenced on the HiSeq platform were used to close gaps in the assembly with IMAGE [59] and correct errors in the assembly using ICORN [60]. All genomes reached improved high quality draft status [58]. Accessory genome components from strains sequenced only as Illumina libraries were assembled using Velvet [61] as described previously [32].

Genome annotation was based on a manually curated transfer and extension of the annotation of *S. pneumoniae* ATCC 700669 [31] using Glimmer3 [62], Artemis and ACT [63].

All other isolates, representing an international collection from Europe and the USA, were sequenced as multiplexed libraries using the Illumina Genome Analyzer II as described previously [32]. All isolates were sequenced to a mean depth of at least 55-fold coverage.

All data have been submitted to the ENA nucleotide sequence database with accession codes listed in Table S1.

### Phylogenetic analysis

To produce the whole genome alignment, for those genomes sequenced using a combination of 454 and capillary technologies, Illumina read pairs of length and insert size the same as the other taxa were simulated from the assembled contigs. Reads were mapped to the reference genome using SMALT, and polymorphisms identified from the resulting alignment, as described previously [64]. An iterative algorithm was used to identify recombinations and generate a phylogeny as described previously [32].

Analysis of the complete whole genome alignment was also performed using BRATnextgen [33]. Based on the maximum likelihood phylogeny's structure, the number of groups was pre-specified as four (clades I, II, III and isolate BHN609). A window size of 1 kb was used and the alpha parameter was learned from the analysis. The threshold for identifying recombinations was exceeding a *p* value of 0.05 based on 100 permutations.

To test whether there was any sign of a molecular clock in the data, for the clade I isolates for which a precise year of isolation was available (*n* = 66), this value was plotted against the root-to-tip distance within the clade. This revealed a significant correlation (*R* ^2^ = 0.07, *p* value = 0.016; Figure S10) and suggested that clade I originated around 1946. Therefore an analysis with BEAST [65] was performed using the alignment of polymorphic sites, which excludes substitutions introduced by putative recombination events, associated with the fixed topology of the tree as displayed in Figure 1. The dates of isolation listed in Table S1 were used to estimate the phylodynamics with a relaxed lognormal mutation rate [66], a skyline plot prior for demographic history [67] and a GTR model of nucleotide substitution with a single rate category. Phylogeographic analysis using discrete locations was conducted using a continuous time Markov chain [68]. Ten chains of 100 million generations were used in the analysis; 50 million generations of each were removed as burnin, and the remainder of the data used to estimate the values described in the test. The ESS values were above 200 for all outputs of the model. The reported substitution rate represents the arithmetic mean of the rate on each branch of the phylogeny.

### Distribution of the accessory genome

The components of the accessory genome were manually identified through comparisons using MUGSY [69] and ACT [63]. To generate heatmaps, the Illumina sequence reads were mapped against these accessory genome sequences using BWA [70], then coverage plots were generated using Samtools [71]. Each sample's plot was standardised according to the corresponding number of reads mapped in the analysis to make them comparable. Along with this general search for all accessory genome loci, a search for sequence polymorphisms within genes associated with resistance, specifically *pbp1a*, *pbp2x*, *pbp2b*, *folP*, *dyr*, *parC*, *parE*, *gyrA* and *gyrB*, was conducted. This failed to identify any variation that has been associated with antibiotic resistance.

### Microarray experiments

Preparation and hybridisation of RNA samples to the BμG@S SPv1.1.0 microarray were completed as described previously [72]. Three independent biological replicates were analyzed for each isolate in the two comparisons (4038 against 4039, and TIGR4 against TIGR4^PUS^) using a common reference experimental design. The BμG@S SPv1.1.0 array design is available in BμG@Sbase (accession number: A-BUGS-14; http://bugs.sgul.ac.uk/A-BUGS-14) and also ArrayExpress (accession number: A-BUGS-14).

Feature extraction of intensity data was performed using BlueFuse v3.5 (Cambridge BlueGnome). The microarray data were median normalized and statistically analyzed using the BioConductor package limma [73], correcting for multiple testing by applying the Benjamini and Hochberg false discovery rate method. Fully annotated microarray data has been deposited in BμG@Sbase (accession number: E-BUGS-144; http://bugs.sgul.ac.uk/E-BUGS-144) and also ArrayExpress (accession number: E-BUGS-144).

### RNA-seq experiments

RNA-seq sample preparation was conducted as described in [42], with the following modifications. Cells were harvested from 10 mL Brain-Heart Infusion (BHI; Oxoid) cultures at an OD_600_ of 0.6 through centrifugation (2,594 *g*, 10 min) at room temperature, then lysed by treatment with 30 mg mL^−1^ lysosyme (Roche) at room temperature for 15 min. Additionally, no effort was made to deplete rRNA from the samples. Three independent paired biological replicates were performed for both experiments and have been submitted to the ENA under accession numbers ERR015603, ERR015600 and ERR220392 for *S. pneumoniae* 99-4038 and ERR015607, ERR015599 and ERR220393 for *S. pneumoniae* 99-4039, respectively.

Mapping of RNA-seq data to the *S. pneumoniae* 99-4038 genome was performed as described in [28]. Briefly, following mapping with BWA [70], only uniquely mapping reads were used in the calculation of read counts; these values were analysed using DESeq [74]. These read counts were then used in the calculation of RPKM values, which were adjusted for the length of the gene to which unique read mapping was possible. This was ascertained through simulating Illumina reads from the reference genome, which were then self-mapped against the complete sequence using the same algorithm as the RNA-seq mapping. This avoided characterizing repetitive coding sequences as being transcribed at artifactually low levels, as the density of read mapping would otherwise seem abnormally low owing to the segments of the feature across which no reads could be uniquely aligned.

Prediction of RNA secondary structure was performed using RNAfold v1.7.2 from the Vienna package using default settings [75].

### Phenotype microarray and antimicrobial sensitivity analysis

Isolates 4038 and 4039 were compared using Omnilog phenotype microarray plates PM9-20 [76]. Frozen stocks of *S. pneumoniae* 4038 and 4039 were passaged twice on horse blood agar plates (Oxoid) under microaerophilic conditions overnight in order to prevent contamination of assays with glycerol. Colonies were then scraped off plates using sterile cotton swabs and dispensed into IF-0a solution (Biolog) at room temperature to a cell density corresponding to 81% transmittance. For each Omnilog phenotype microarray plate used (PM9-20) [76], 880 µL of this cell suspension was added to 10 mL IF-10b GP/GP solution (Biolog) and 120 µL dye mix G (Biolog). This was then supplemented with a 1 mL solution of 7.5 mM D-ribose (Sigma), 2 mM magnesium chloride, 1 mM calcium chloride, 2 mM sodium pyrophosphate (Sigma), 25 µM L-arginine (Sigma), 25 µM L-methionine (Sigma), 25 µM hypoxanthine (Sigma), 10 µM lipoamide (Sigma), 5 µM nicotine adenine dinucleotide (Sigma), 0.25 µM riboflavin (Sigma), 0.005% by mass yeast extract (Fluka) and 0.005% by mass Tween 80 (Sigma). One hundred microliters of this mixture was dispensed into each well on the assay plate. Plates were then allowed to equilibrate in an anaerobic atmosphere (80% N_2_, 10% CO_2_, 10% H_2_) for 5 min prior to being sealed in airtight bags and loaded into the Omnilog machine (Biolog). Plates were scanned every 10 min for 48 h while incubated at 37°C. Two paired replicates were performed for the two isolates.

The data were exported from the Biolog File Manager, and further analysis was conducted in R. The data was transformed in to signal values as described previously [77]. The Bioconductor package limma [73] was used to examine differential metabolism between isolates. *S. pneumoniae* 99-4038 was used as a baseline from which differences were calculated. Benjamini-Hochberg corrected *p* values were used to determine statistical significance of differences controlling for a false discovery rate of 5%.

Susceptibility to a variety of common antibiotics was also determined using the Vitek 2 analyzer (bioMérieux) and the AST-ST01 Streptococcus susceptibility card (bioMérieux) using a starting inoculum density of 0.5 McFarland standard as measured by densitometer (bioMérieux). Ciprofloxacin susceptibility was determined using an E test (bioMérieux). An inoculum density of 0.5 McFarland standard was grown on Mueller Hinton Agar with 5% sheep blood (Oxoid) and incubated at 37°C in a 6% CO_2_ atmosphere for 20 h.

### Mouse model of disease

Female outbred MF1 mice were purchased from Harlan Olac, Bicester, UK. For pneumonia infection 9 week old mice were lightly anaesthetised by inhalation of 2.5% v/v fluorothane. Viable pneumococci at CFU/mL as indicated were then administered via the nostrils in a 50 mL volume. Signs of disease were monitored frequently until mice were deemed to have irreversibly succumbed to the infection [78]; they were then humanely sacrificed through exposure to carbon dioxide followed by cervical dislocation. Cardiac blood was sampled immediately, and then skin and muscles above the trachea were separated and the trachea was dissected with microscissors. A fine tipped sterile Pasteur pipette was inserted into each nostril in turn and 1 ml of tissue culture grade PBS was pipetted through then collected from trachea. Approximately 1 mL of nasopharyngeal lavage fluid (NPL) was recovered from each mouse. No perfusion of the circulatory system with PBS was performed as previous work has found the contribution of blood borne bacteria to organ counts not to be significant. Organs harvested for bacteriological investigation were suspended in 2–5 mL PBS as appropriate and homogenized. Viable bacteria in blood, NPL, and tissues were counted by plating out serial dilutions on blood agar base number 2 plus 5% v/v horse blood.

### Construction and analysis of *S. pneumoniae* TIGR4^PUS^

The region upstream of *patAB* in *S. pneumoniae* 99-4038 and 99-4039 was amplified through PCR using primers IntGL (GCCTGCCACTTGTAGGTTTT) and IntGR (GATAGGGCAGAAGAGCATCC). The ∼500 bp PCR products were each purified through agarose gel electrophoresis using a QIAquick Gel Extraction Kit (Qiagen) and then ligated into pGEM-T Easy (Promega) using T4 ligase (Promega) in a 10 µL reaction volume, according to manufacturer's instructions. A 1 µL sample of this reaction was then used to transform electrocompetent *E. coli* TOP10 cells (Invitrogen) through electroporation with a 2.5 kV pulse. These cells were then grown in 250 µL SOC medium (Invitrogen), shaken at 37°C for 2 h. A 50 µL sample of this culture was then spread on Luria broth (LB) agar plates supplemented with 100 µg mL^−1^ ampicillin (Sigma), 300 µg mL^−1^ S-Gal (Sigma) and 30 µg mL^−1^ isopropyl β-thiogalactoside (Sigma). White colonies were then picked, grown in LB supplemented with 100 µg mL^−1^ ampicillin and stored. The sequences of the plasmid inserts were then amplified by PCR and checked through capillary sequencing. Both plasmids were then extracted from their host *E. coli* using the QIAprep Spin Miniprep Kit (Qiagen) and diluted to 25 µg mL^−1^. These two stocks were then used to transform three *S. pneumoniae* TIGR4 cultures in parallel as described in [32]. After 2 h growth, a 50 µL sample of each transformation reaction was used to inoculate either BHI or BHI supplemented with 2 µg mL^−1^ ciprofloxacin. Colonies were then isolated from the *S. pneumoniae* TIGR4 culture transformed with the region upstream of *patAB* from *S. pneumoniae* 99-4039 after 20 h growth in Brain-Heart Infusion (Oxoid) supplemented with 2 mg L^−1^ ciprofloxacin. PCR amplification and sequencing of the region upstream of *patAB* in three colonies from each of the three transformation experiments revealed they all shared the PUS; one of these was stored and designated *S. pneumoniae* TIGR4^PUS^. A negative control transformation with the region upstream of *patAB* from *S. pneumoniae* 99-4038, conducted in parallel, did not yield a similarly high density of fluoroquinolone-resistant colonies.

## Supporting Information

Figure S1 Distribution of homologous recombination lengths. This histogram shows the lengths of the 79 detected recombinations that occur outside of the annotated mobile genetic elements in the OXC141 reference genome. These fit an exponential length distribution with a rate parameter of 8.71×10^−5^ bp^−1^ (95% confidence interval of 7.15×10^−5^–1.10×10^−4^ bp^−1^), which is indicated on the plot by the red curve.
(PDF)

Figure S2 Analysis of sequence using BRATnextgen. (A) The phylogeny of the lineage, and (B) the annotation of the reference genome, are displayed as in Figure 1. (C) The coloured bars underneath the annotation indicate an independent analysis of sequence exchange within the lineage, performed using BRATnextgen. Each of the bars represents the cluster to which the sequence belongs at different points along the genome; the key indicates the groups to which the colours correspond. The analysis confirms that there is little sequence exchange within clade I, in which the sequence almost entirely belongs to group 1. The only significant recombination is a block from the ‘outgroup’ (i.e. a genotype outside the collection) representing the acquisition of prophage **φ**BHN167, in agreement with the analysis displayed in Figure 1. No substantial exchange of sequence between clades is observed anywhere in the collection, with the only large recombination detected being an apparent exchange of sequence within clade II.
(PDF)

Figure S3 Heatmap showing the distribution of prophage sequences between serotype 3 isolates. (A) All available *S. pneumoniae* prophage genomes, including φOXC141, φBHN167 (ENA accession code HF563658) and φ072838 derived from genomes sequenced as part of this study, are clustered and grouped based on gene content as described in [29]. The alternating orange and brown bars underneath the clustering indicate the extent of each prophage. (B) The maximum likelihood phylogeny of the serotype 3 isolates is displayed as in Figure 1. (C) A heatmap of Illumina sequence read coverage of the prophage sequences per million reads mapped is displayed for each taxon. Blue indicates low coverage, and red indicates high coverage.
(PDF)

Figure S4 Activity of prophage sequences. (A) Annotation of prophage φOXC141 and the flanking genomic regions. The modular organisation typical of pneumococcal prophage is marked relative to the viral sequence. (B) Mapping of Illumina genome sequencing data from isolates (i) BHN640 (ii) BHN035 and (iii) BHN598, phylogenetically disparate within clade I. The two- to three-fold increased coverage of φOXC141 suggests it is actively replicating. (C) Prophage transcription. The RNA-seq data from (i) 4038 and (ii) 4039 indicate that in some cells, the lysogeny module is active, keeping the prophage dormant, while in others, the modules required for replication and host cell lysis are expressed.
(PDF)

Figure S5 Histogram showing the genome-wide levels of protein CDS expression. The distribution of coding sequence read counts per kilobase length per million reads mapped (RPKM) values, on a base 10 logarithmic scale, is shown as a histogram. This shows the continuum of expression levels observed in the data, with no evidence of any set of discrete expression levels.
(PDF)

Figure S6 Boxplots showing the mean level of transcription of CDSs, as grouped according to functional annotation. This shows the group of proteins most highly transcribed, on average, are those involved in the core pathways of energy metabolism and nucleic acid and protein synthesis. Pseudogenes tend to exhibit lower levels of transcription than functional genes.
(PDF)

Figure S7 Scatterplot relating the level of gene expression to the relative amount of antisense transcription according to gene category. Each point plots the base 10 logarithm of the level of sense transcription against the base 10 logarithm of the ratio of sense to antisense transcription. Each point is coloured according to its function as in Figure S6, with the exception of the pathogenicity, adaptation and chaperone proteins, which are coloured black. Two cases are evident of genes with putative functional information being transcribed in the antisense direction at a high level. One is the *comCDE* operon, which encodes the competence stimulating peptide precursor and its cognate receptor, and the other is the SP4038_7830-SP4038_07880 operon, encoding a restriction modification system. Both are marked on the plot.
(PDF)

Figure S8 Construction of *S. pneumoniae* TIGR4^PUS^. A sample of *S. pneumoniae* TIGR4 was transformed with the region upstream of *patAB* from either (A) and (C) *S. pneumoniae* 99-4038 or (B) and (D) *S. pneumoniae* 99-4039. The transformed cultures were then grown in (A) and (B) BHI or (C) and (D) BHI supplemented with 2 µg mL^−1^ ciprofloxacin. The bars show the mean optical density at 600 nm after 20 h growth at 37°C, with the error bars showing one standard error of the mean. *S. pneumoniae* TIGR4^PUS^ was isolated after culturing isolates from (D) on horse blood agar plates supplemented with 2 µg mL^−1^ ciprofloxacin.
(PDF)

Figure S9 Further *in vivo* experiments comparing isolates *S. pneumoniae* TIGR4 and TIGR4^PUS^, where the latter has had the PUS introduced *in vitro*. The outcomes displayed are: (A) survival curves, (B) level of bacteraemia (C) counts of the two isolates in different organs following intraperitoneal inoculation with 10^4^ cfu. In Figure 4, it is shown that *S. pneumoniae* 4039 kills all mice significantly quicker than *S. pneumoniae* 4038, although there was not a significant difference *between S. pneumoniae* TIGR4 and TIGR4^PUS^ by the same metric. *S. pneumoniae* TIGR4^PUS^ is found at a significantly higher density in the mouse brain relative to TIGR4.
(PDF)

Figure S10 Root-to-tip plot showing genetic divergence of clade I over time. For the 66 samples within clade I for which a precise year of isolation was available, this value was plotted against the distance of each isolate from the root of the clade, as according to the maximum likelihood tree displayed in Figure 1. This reveals a weak positive correlation that suggests that the clade originated around 1946.
(PDF)

Table S1 Epidemiological data and ENA accession codes for all sequences used in the phylogenomic analysis.
(XLSX)

Table S2 Polymorphisms distinguishing *S. pneumoniae* 99-4038 and 99-4039.
(DOCX)

Table S3 Significant differences in expression patterns between *S. pneumoniae* 4038 and 4039 detected using a microarray based on the *S. pneumoniae* TIGR4 and R6 genomes. Statistical analysis was performed using limma. The displayed *p* value is adjusted to reflect correction for multiple testing using the Benjamini-Hochberg method.
(DOCX)

Table S4 Significant differences in expression patterns between *S. pneumoniae* 4038 and 4039 detected using a RNA-seq. Statistical analysis was performed using DESeq. The displayed *p* value is adjusted to reflect correction for multiple testing using the Benjamini-Hochberg method.
(DOCX)

Table S5 Results of the Omnilog phenotype microarray assay. This table lists the compounds found to result in significantly higher respiration in 4039 relative to 4038. Each compound was tested at four different concentrations; that concentration at which a significant difference was observed is indicated by a number, with one being the lowest concentration and four being the highest. The displayed *p* value is adjusted to reflect correction for multiple testing using the Benjamini-Hochberg method.
(DOCX)

Table S6 Susceptibility of isolates *S. pneumoniae* 99-4038, 99-4039, TIGR4 and TIGR4^PUS^ to common antibiotics. Minimum inhibitory concentrations (MICs) were determined either used the Vitek 2 or E test methods.
(DOCX)

Table S7 Significant differences in expression patterns between *S. pneumoniae* TIGR4 and the modified strain carrying the PUS (TIGR4^PUS^), detected using a microarray based on the *S. pneumoniae* TIGR4 genome. Statistical analysis was performed using limma. The displayed *p* value is adjusted to reflect multiple testing using the Benjamini-Hochberg method.
(DOCX)
