# Supplementary figures and images for: Dominant Role of Nucleotide Substitution in the Diversification of Serotype 3 Pneumococci over Decades and during a Single Infection
Source: PLoS Genet. 2013 Oct 10;9(10):e1003868. doi: 10.1371/journal.pgen.1003868 (PMC3794909; doi:10.1371/journal.pgen.1003868)

Figure S1

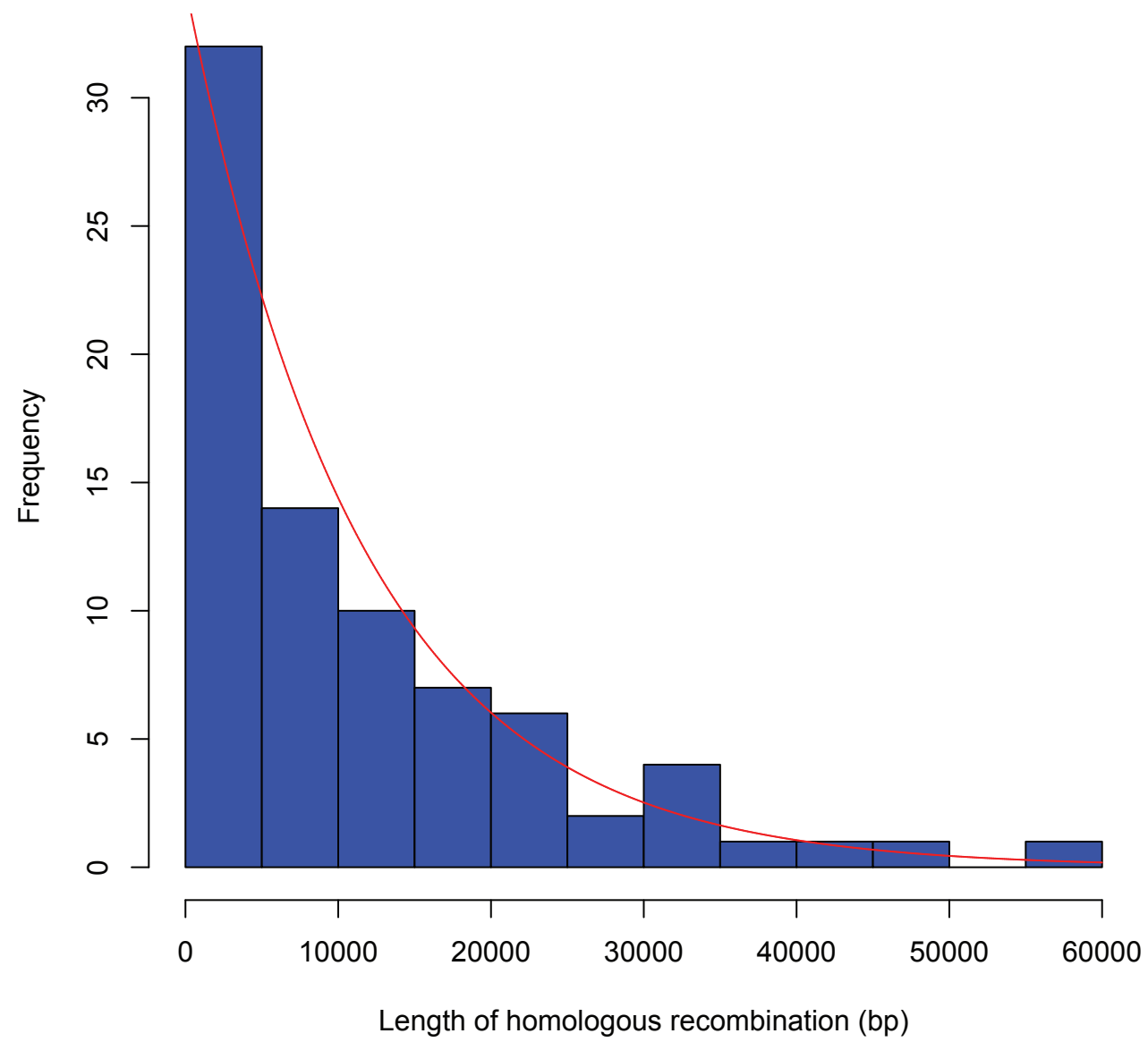

Supplement: Figure S1 — Distribution of homologous recombination lengths. This histogram shows the lengths of the 79 detected recombinations that occur outside of the annotated mobile genetic elements in the OXC141 reference genome. These fit an exponential length distribution with a rate parameter of 8.71×10−5 bp−1 (95% confidence interval of 7.15×10^−5^–1.10×10−4 bp−1), which is indicated on the plot by the red curve. (PDF) [file pgen.1003868.s001.pdf]

Figure S2

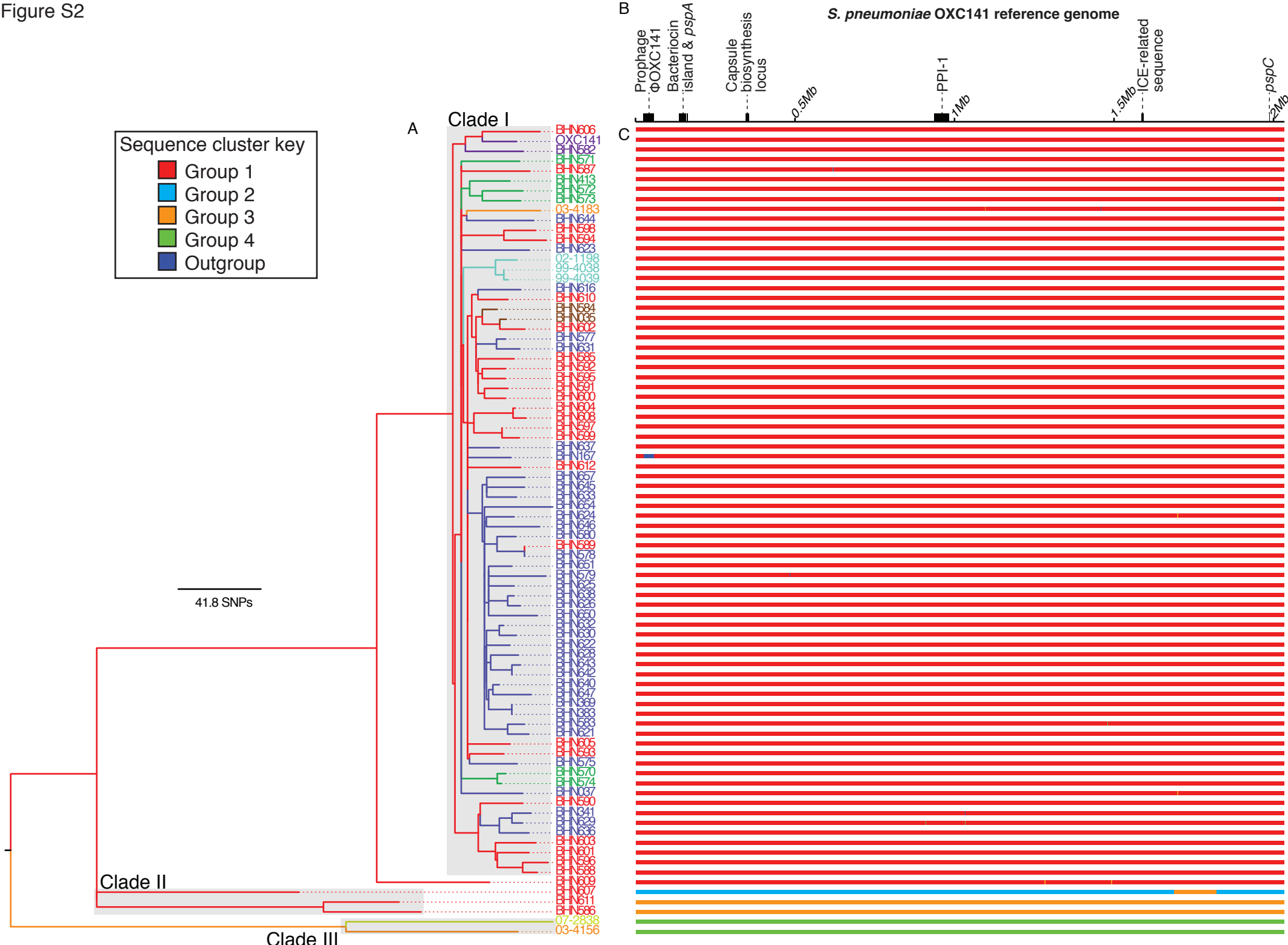

Supplement: Figure S2 — Analysis of sequence using BRATnextgen. (A) The phylogeny of the lineage, and (B) the annotation of the reference genome, are displayed as in Figure 1. (C) The coloured bars underneath the annotation indicate an independent analysis of sequence exchange within the lineage, performed using BRATnextgen. Each of the bars represents the cluster to which the sequence belongs at different points along the genome; the key indicates the groups to which the colours correspond. The analysis confirms that there is little sequence exchange within clade I, in which the sequence almost entirely belongs to group 1. The only significant recombination is a block from the ‘outgroup’ (i.e. a genotype outside the collection) representing the acquisition of prophage φBHN167, in agreement with the analysis displayed in Figure 1. No substantial exchange of sequence between clades is observed anywhere in the collection, with the only large recombination detected being an apparent exchange of sequence within clade II. (PDF) [file pgen.1003868.s002.pdf]

Figure S3

B

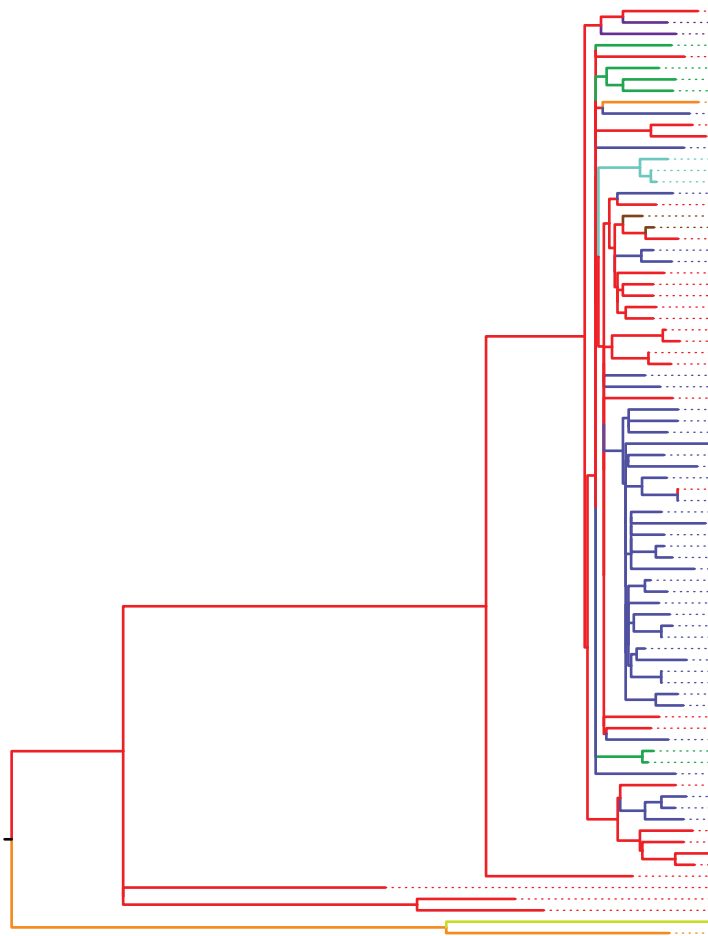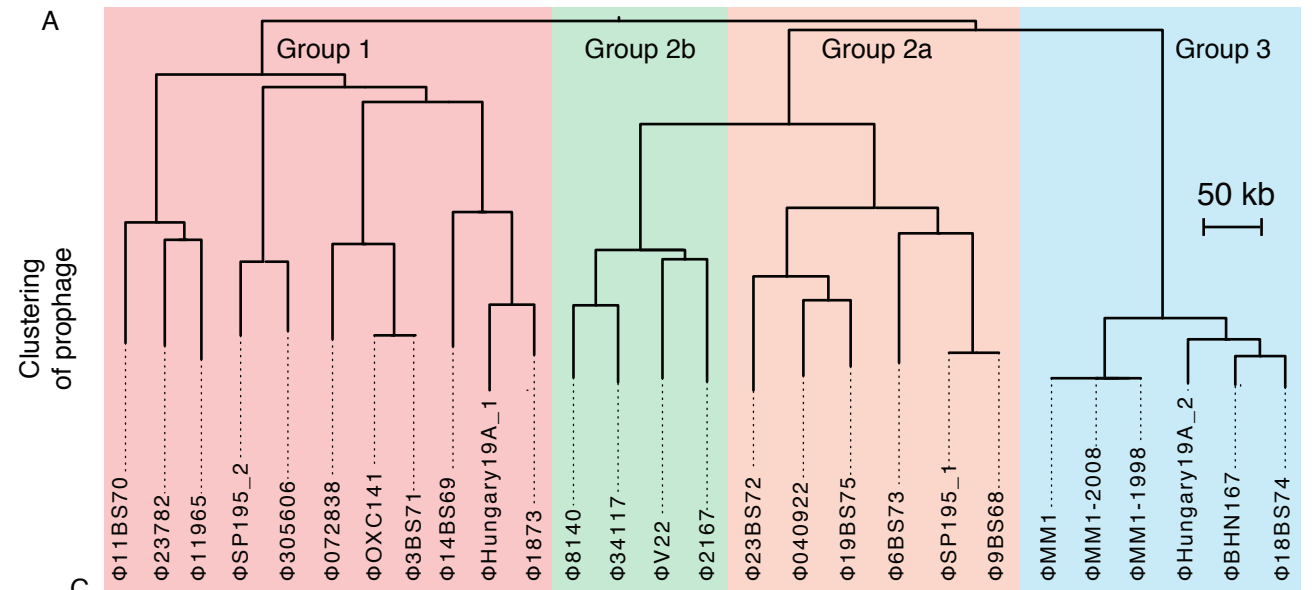

C

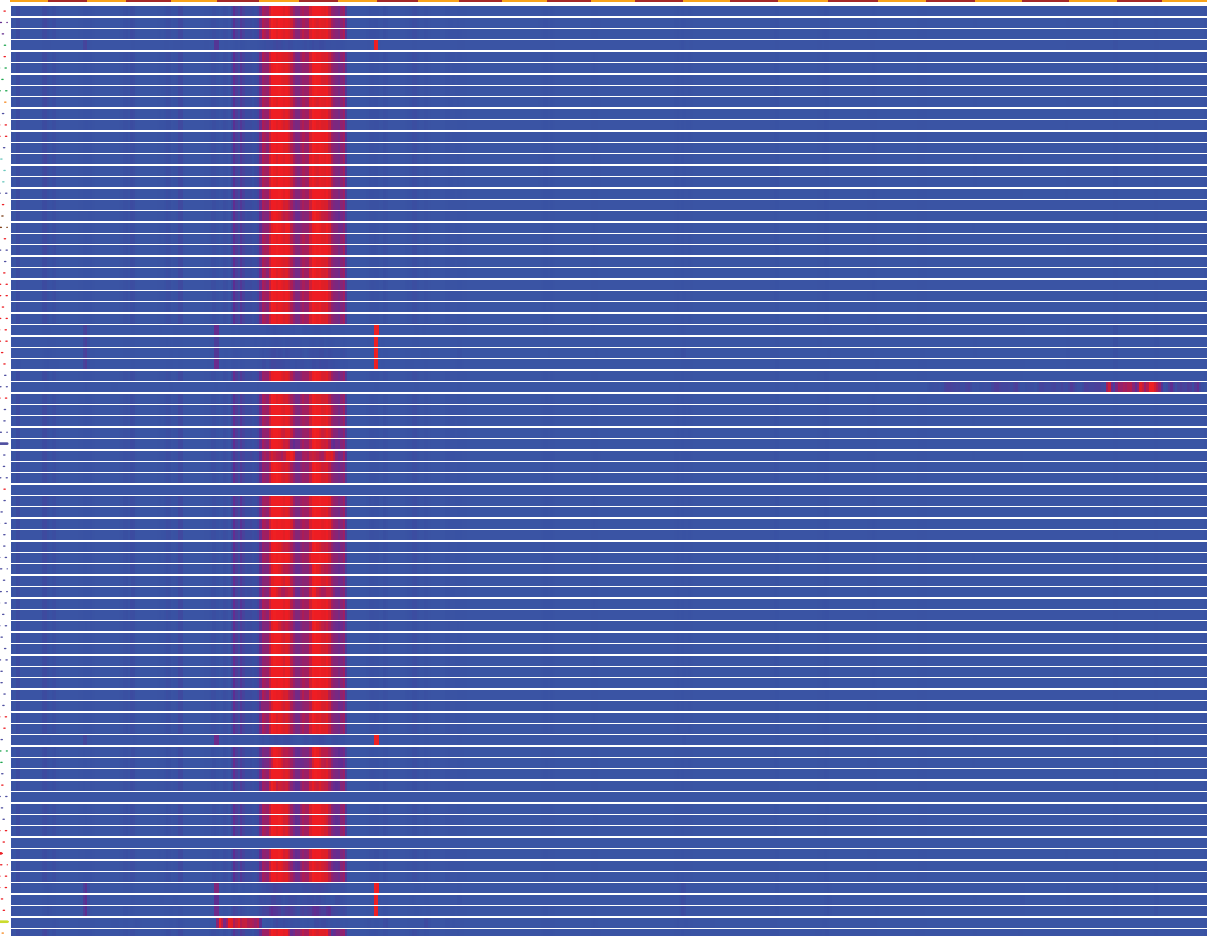

Supplement: Figure S3 — Heatmap showing the distribution of prophage sequences between serotype 3 isolates. (A) All available S. pneumoniae prophage genomes, including φOXC141, φBHN167 (ENA accession code HF563658) and φ072838 derived from genomes sequenced as part of this study, are clustered and grouped based on gene content as described in [29]. The alternating orange and brown bars underneath the clustering indicate the extent of each prophage. (B) The maximum likelihood phylogeny of the serotype 3 isolates is displayed as in Figure 1. (C) A heatmap of Illumina sequence read coverage of the prophage sequences per million reads mapped is displayed for each taxon. Blue indicates low coverage, and red indicates high coverage. (PDF) [file pgen.1003868.s003.pdf]

Figure S4

A

Prophage  $\Phi$ OXC141

10 kb

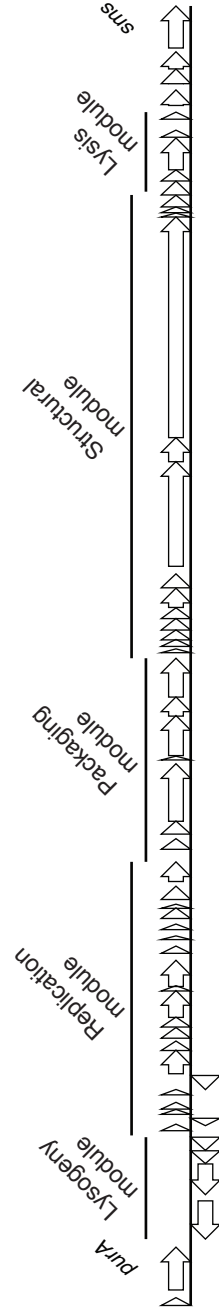

B

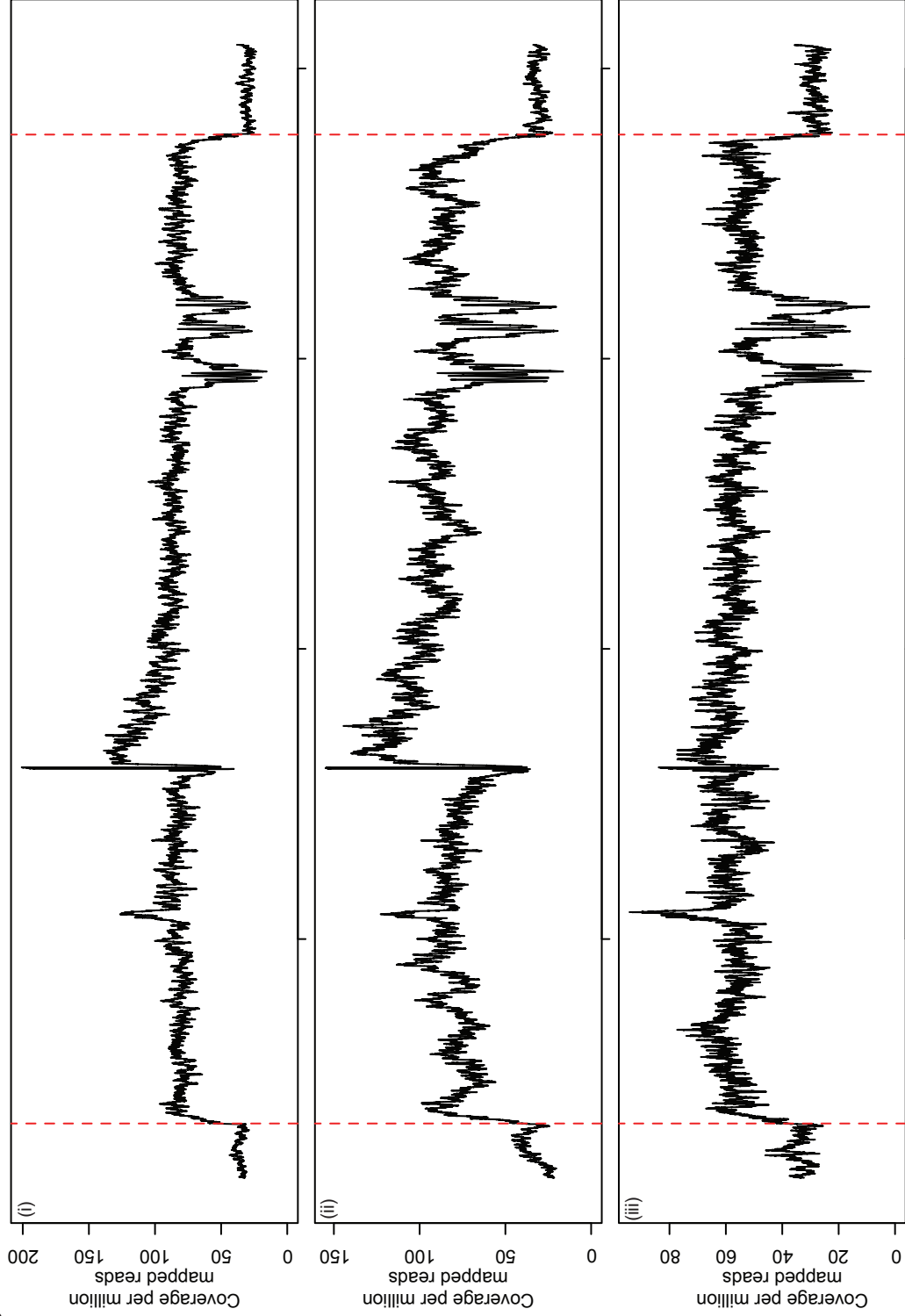

C

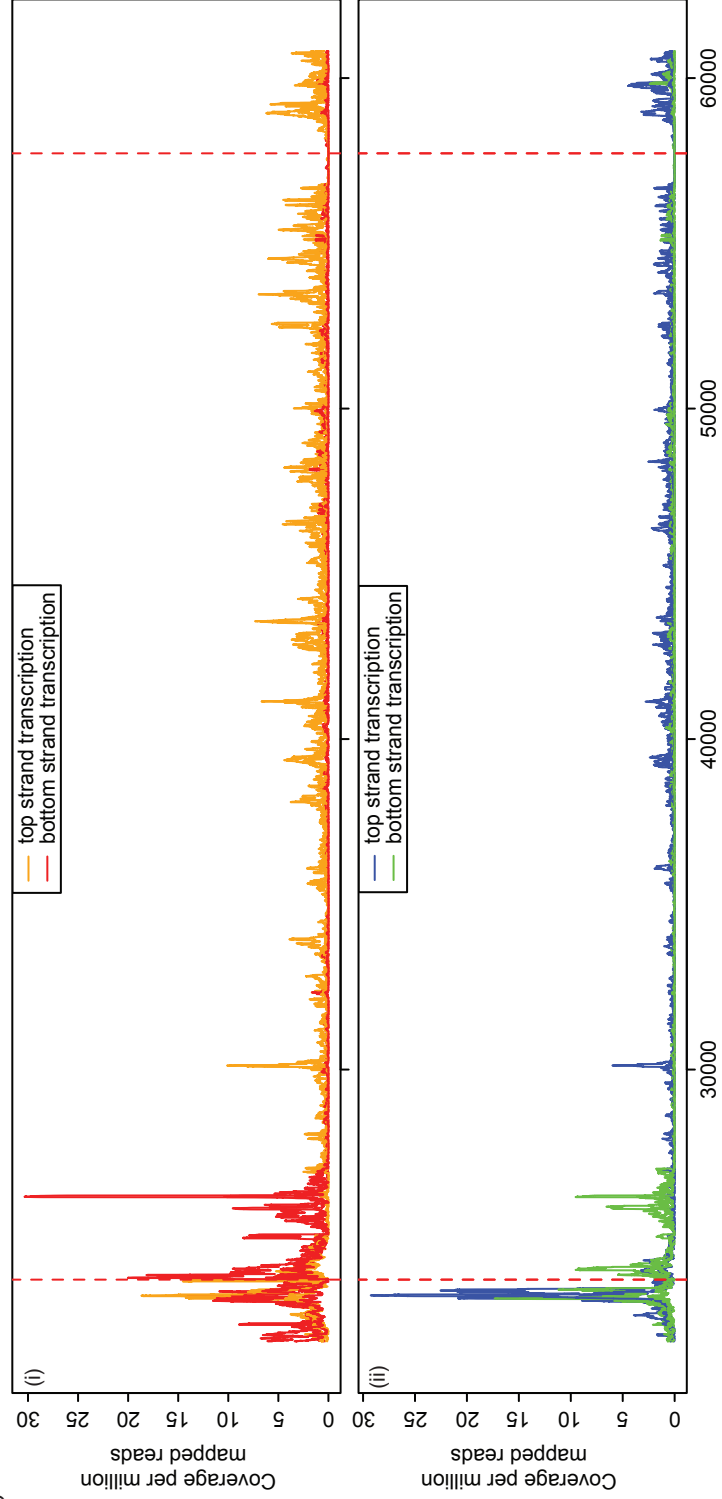

Supplement: Figure S4 — Activity of prophage sequences. (A) Annotation of prophage φOXC141 and the flanking genomic regions. The modular organisation typical of pneumococcal prophage is marked relative to the viral sequence. (B) Mapping of Illumina genome sequencing data from isolates (i) BHN640 (ii) BHN035 and (iii) BHN598, phylogenetically disparate within clade I. The two- to three-fold increased coverage of φOXC141 suggests it is actively replicating. (C) Prophage transcription. The RNA-seq data from (i) 4038 and (ii) 4039 indicate that in some cells, the lysogeny module is active, keeping the prophage dormant, while in others, the modules required for replication and host cell lysis are expressed. (PDF) [file pgen.1003868.s004.pdf]

Figure S5

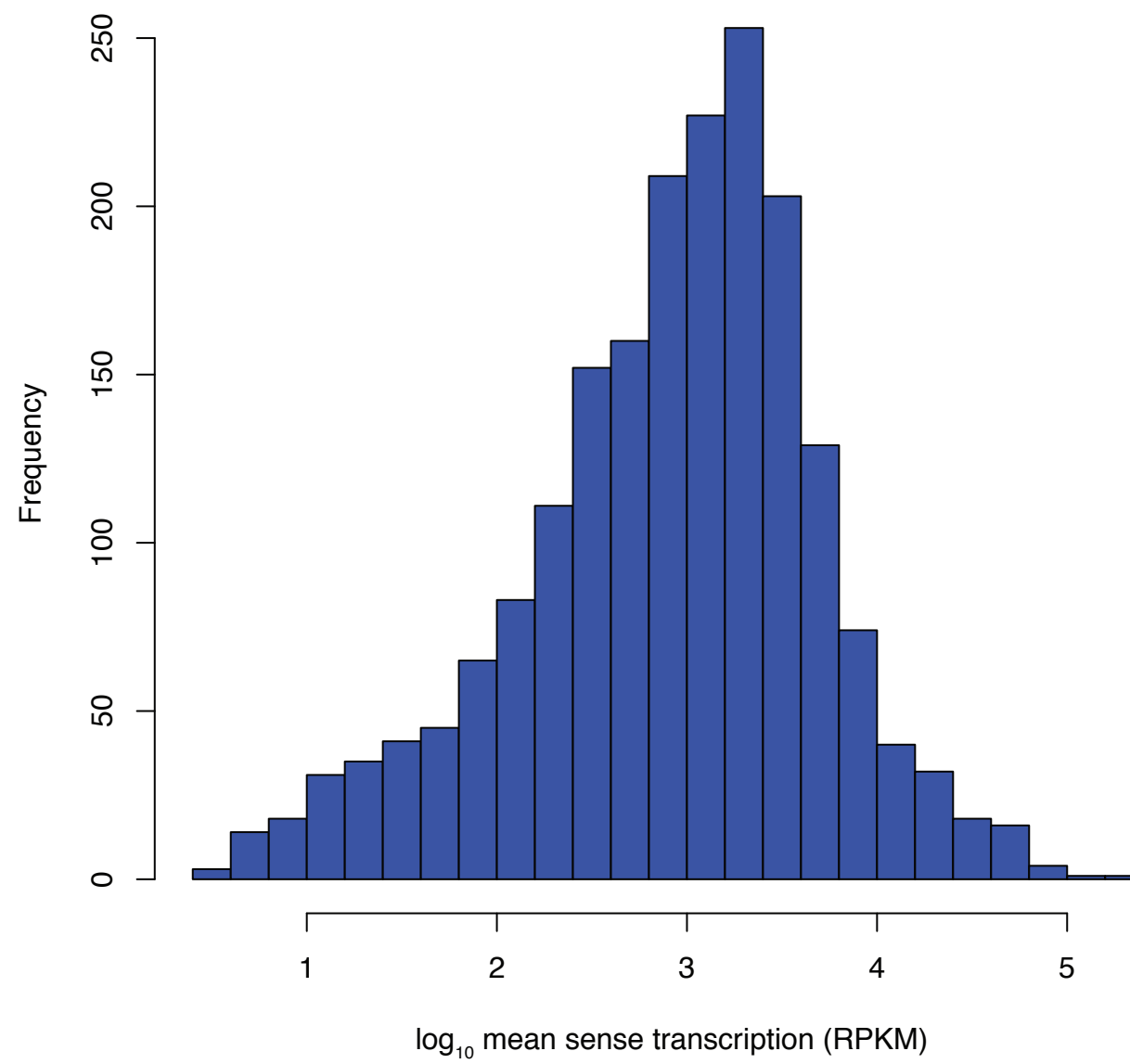

Supplement: Figure S5 — Histogram showing the genome-wide levels of protein CDS expression. The distribution of coding sequence read counts per kilobase length per million reads mapped (RPKM) values, on a base 10 logarithmic scale, is shown as a histogram. This shows the continuum of expression levels observed in the data, with no evidence of any set of discrete expression levels. (PDF) [file pgen.1003868.s005.pdf]

Figure S6

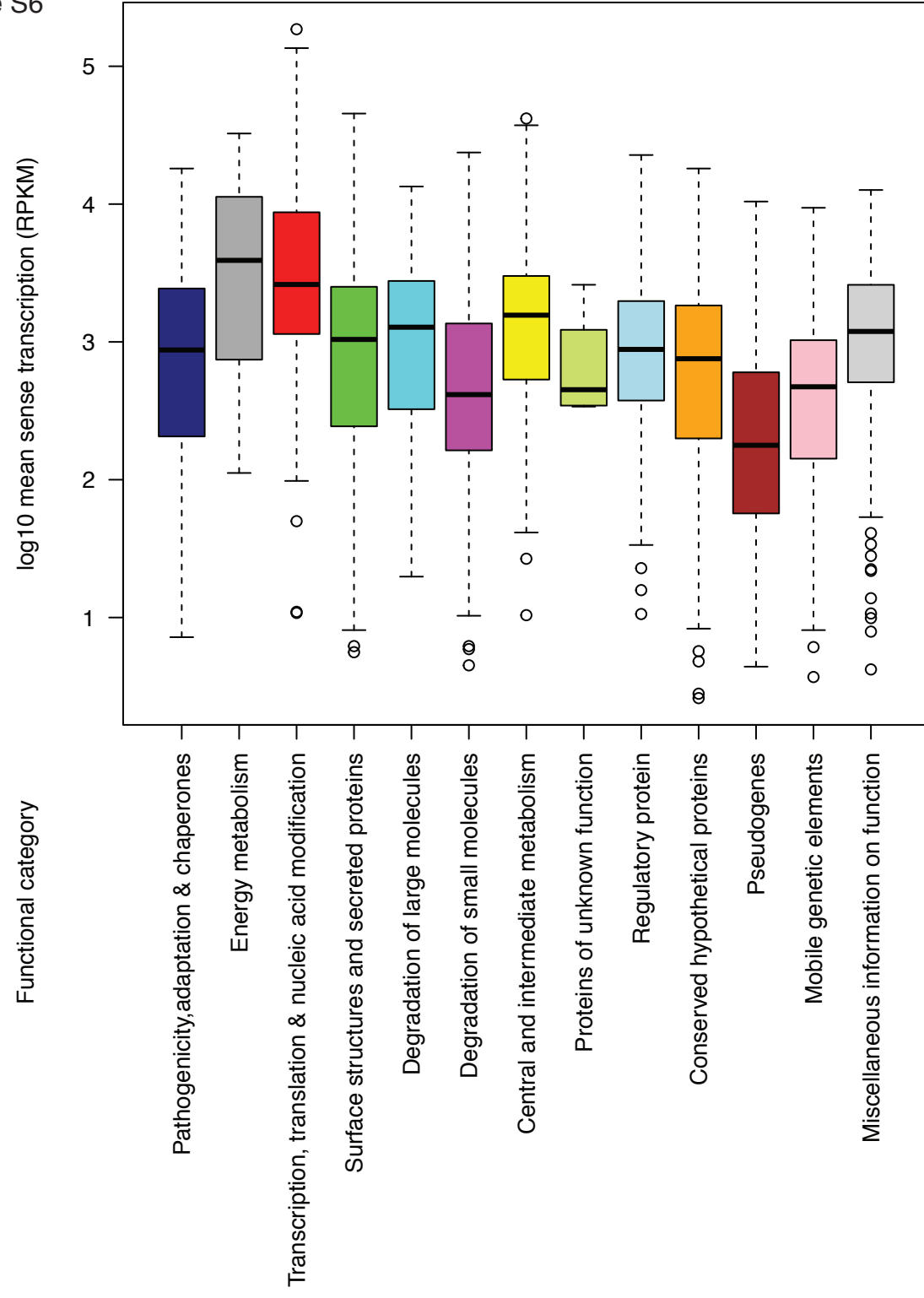

Supplement: Figure S6 — Boxplots showing the mean level of transcription of CDSs, as grouped according to functional annotation. This shows the group of proteins most highly transcribed, on average, are those involved in the core pathways of energy metabolism and nucleic acid and protein synthesis. Pseudogenes tend to exhibit lower levels of transcription than functional genes. (PDF) [file pgen.1003868.s006.pdf]

Figure S7

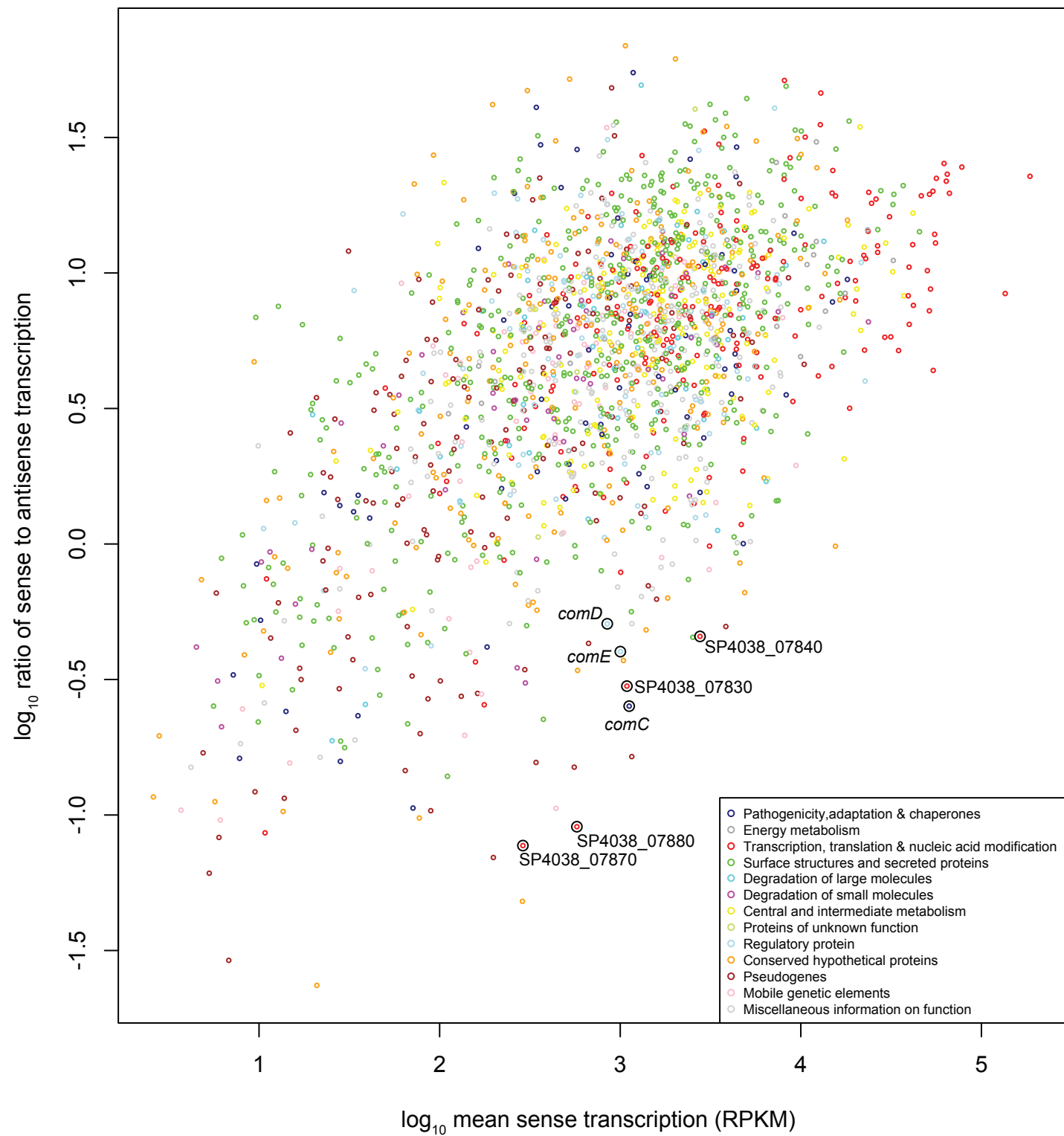

Supplement: Figure S7 — Scatterplot relating the level of gene expression to the relative amount of antisense transcription according to gene category. Each point plots the base 10 logarithm of the level of sense transcription against the base 10 logarithm of the ratio of sense to antisense transcription. Each point is coloured according to its function as in Figure S6, with the exception of the pathogenicity, adaptation and chaperone proteins, which are coloured black. Two cases are evident of genes with putative functional information being transcribed in the antisense direction at a high level. One is the comCDE operon, which encodes the competence stimulating peptide precursor and its cognate receptor, and the other is the SP4038_7830-SP4038_07880 operon, encoding a restriction modification system. Both are marked on the plot. (PDF) [file pgen.1003868.s007.pdf]

Figure S8

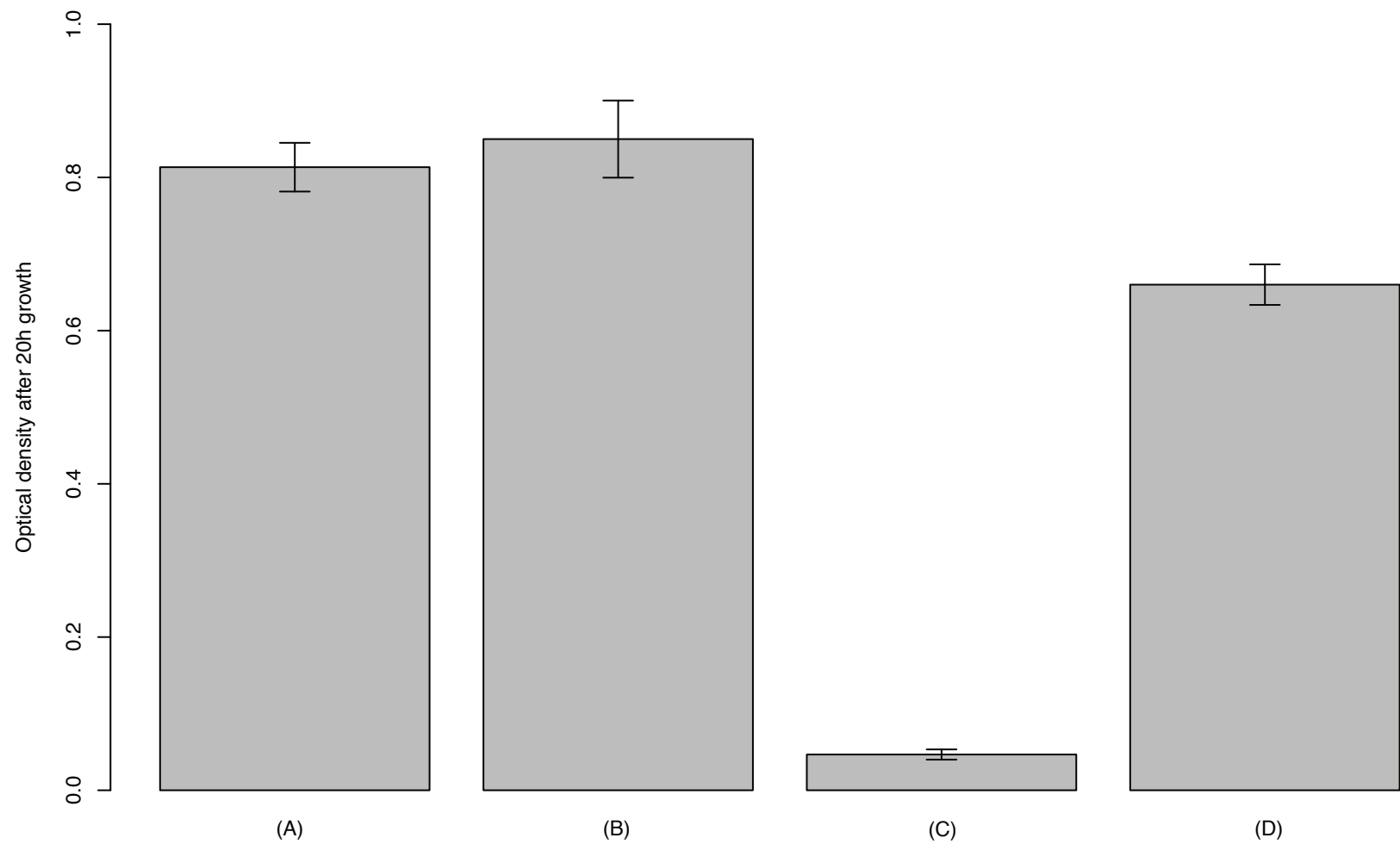

Supplement: Figure S8 — Construction of S. pneumoniae TIGR4PUS. A sample of S. pneumoniae TIGR4 was transformed with the region upstream of patAB from either (A) and (C) S. pneumoniae 99-4038 or (B) and (D) S. pneumoniae 99-4039. The transformed cultures were then grown in (A) and (B) BHI or (C) and (D) BHI supplemented with 2 µg mL−1 ciprofloxacin. The bars show the mean optical density at 600 nm after 20 h growth at 37°C, with the error bars showing one standard error of the mean. S. pneumoniae TIGR4PUS was isolated after culturing isolates from (D) on horse blood agar plates supplemented with 2 µg mL−1 ciprofloxacin. (PDF) [file pgen.1003868.s008.pdf]

Figure S9

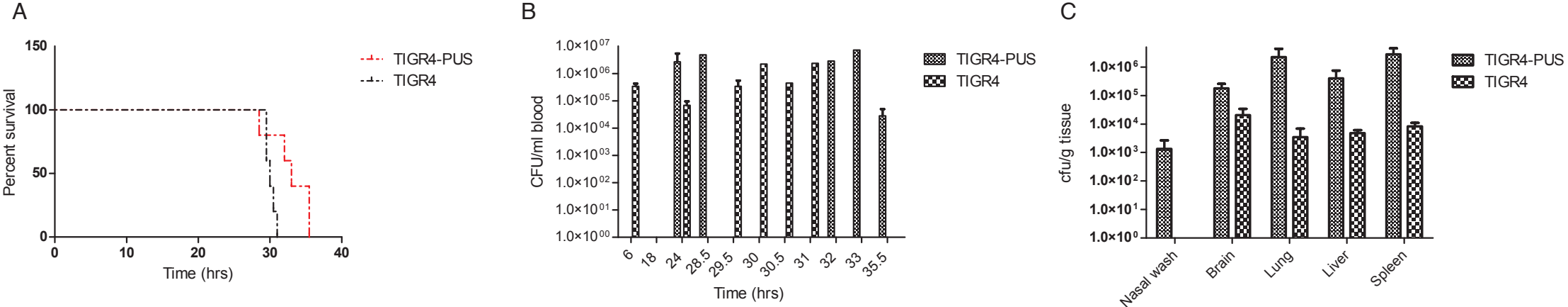

Supplement: Figure S9 — Further in vivo experiments comparing isolates S. pneumoniae TIGR4 and TIGR4PUS, where the latter has had the PUS introduced in vitro. The outcomes displayed are: (A) survival curves, (B) level of bacteraemia (C) counts of the two isolates in different organs following intraperitoneal inoculation with 104 cfu. In Figure 4, it is shown that S. pneumoniae 4039 kills all mice significantly quicker than S. pneumoniae 4038, although there was not a significant difference between S. pneumoniae TIGR4 and TIGR4PUS by the same metric. S. pneumoniae TIGR4PUS is found at a significantly higher density in the mouse brain relative to TIGR4. (PDF) [file pgen.1003868.s009.pdf]

Figure S10

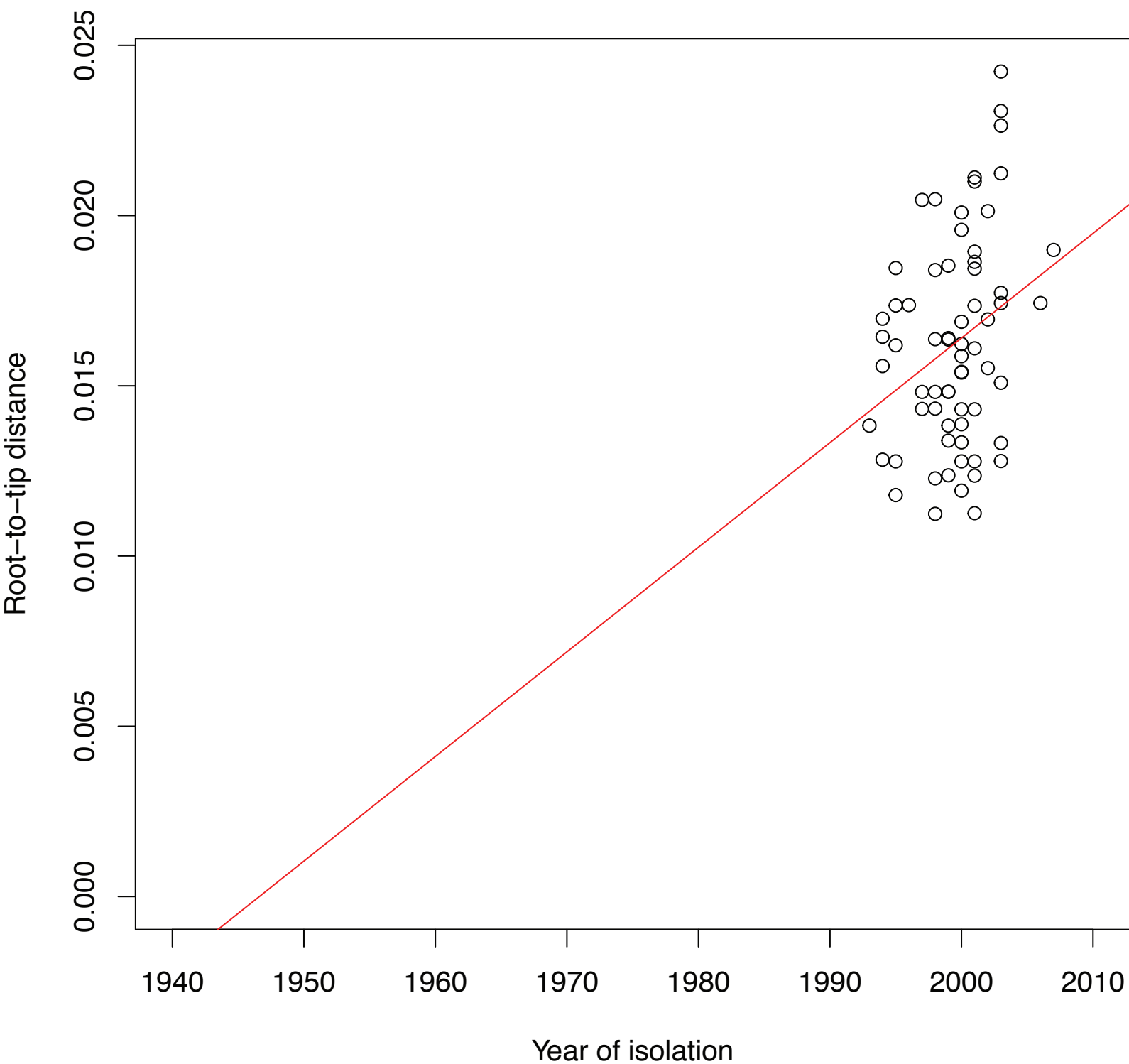

Supplement: Figure S10 — Root-to-tip plot showing genetic divergence of clade I over time. For the 66 samples within clade I for which a precise year of isolation was available, this value was plotted against the distance of each isolate from the root of the clade, as according to the maximum likelihood tree displayed in Figure 1. This reveals a weak positive correlation that suggests that the clade originated around 1946. (PDF) [file pgen.1003868.s010.pdf]
