# Supplementary material for: Dominant Role of Nucleotide Substitution in the Diversification of Serotype 3 Pneumococci over Decades and during a Single Infection
Source: PLoS Genet. 2013 Oct 10;9(10):e1003868. doi: 10.1371/journal.pgen.1003868 (PMC3794909; doi:10.1371/journal.pgen.1003868)
Supplement: Table S2 — Polymorphisms distinguishing S. pneumoniae 99-4038 and 99-4039. (DOCX) [file pgen.1003868.s012.docx]

**Table S2**

| **Mutation Location in 4038** | **Effect** | **Locus** | **Comment** |
| --- | --- | --- | --- |
| Base substitution C114255T | Synonymous substitution in surface protein SP4038_01180/SP4039_01180 | Repetitive surface protein | Likely misassembly |
| Base substitution C114420T | Synonymous substitution in surface protein SP4038_01180/SP4039_01180 | Repetitive surface protein | Likely misassembly |
| 31 bp deletion in 4038 at 460183 | Rearrangement of *hsdS* locus | Hypervariable *hsdS* locus | Likely *in vitro* variation |
| 15 bp deletion in 4039 between 460183 and 460197 | Rearrangement of *hsdS* locus | Hypervariable *hsdS* locus |  |
| 15 bp deletion in 4038 at 461251 | Rearrangement of *hsdS* locus | Hypervariable *hsdS* locus | Likely *in vitro* variation |
| 15 bp deletion in 4039 between 461251 and 461265 | Rearrangement of *hsdS* locus | Hypervariable *hsdS* locus | Likely *in vitro* variation |
| 16 bp deletion in 4039 between 462723 and 462738 | Rearrangement of *hsdS* locus | Hypervariable *hsdS* locus | Likely *in vitro* variation |
| Base substitution G836817A | Synonymous substitution in regulatory protein SP4038_08190/SP4039_08200 | Non-repetitive CDS | High-confidence polymorphism |
| Base substitution T894101C | Synonymous substitution in IS element pseudogene SP4038_08850/SP4039_08860 | Repeat structure assembled from generic reads in 4039 | Likely misassembly |
| Base substitution C1177311A | L227M amino acid substitution in putative hydrolase SP4038_11450/SP4039_11460 | Non-repetitive CDS | High-confidence polymorphism |
| 11 bp deletion in 4039 between 1410204 and 1410214 | Truncation of IS element SP4038_13860/SP4039_13870 | Repeat structure assembled from generic reads in 4039 | Likely misassembly |
| Base substitution C1410257T | M76I amino acid substitution in IS element SP4038_13860/SP4039_13870 | Repeat structure assembled from generic reads in 4039 | Likely misassembly |
| 1 bp deletion in 4039 at 1410294 | Truncation of IS element SP4038_13860/SP4039_13870 | Repeat structure assembled from generic reads in 4039 | Likely misassembly |
| 1 bp deletion in 4038 at 1410321 | Truncation of IS element SP4038_13860/SP4039_13870 | Repeat structure assembled from generic reads in 4039 | Likely misassembly |
| Base substitution G1410401A | Synonymous substitution in IS element SP4038_13860/SP4039_13870 | Repeat structure assembled from generic reads in 4039 | Likely misassembly |
| 75 bp deletion in 4038 at 1410585 | Truncation of IS element SP4038_13870/SP4039_13880 | Repeat structure assembled from generic reads in 4039 | Likely misassembly |
| 2 bp deletion in 4038 at 1410589 | Truncation of IS element SP4038_13870/SP4039_13880 | Repeat structure assembled from generic reads in 4039 | Likely misassembly |
| 1 bp deletion in 4038 at 1550906 | Truncation of putative phosphohydrolase SP4038_15170/SP4039_15180 | Non-repetitive CDS | High-confidence polymorphism |
| Base substitution G1691513A | C50T substitution in 23S rRNA | Repeat structure assembled from generic reads in 4038 | Likely misassembly |
| Base substitution G1782903A | C50T substitution in 23S rRNA | Repeat structure | Likely misassembly |
| 1 bp deletion in 4038 at 1845724 | Loss of G910 from 16S rRNA | Repeat structure assembled from generic reads in 4038 and 4039 | Likely misassembly |
| Base substitution C1855761A | C->A substitution 46 nt upstream of *patA* | Non-repetitive locus | High-confidence polymorphism |
| 198 bp deletion in 4038 at 1976945 | In-frame shortening of PspC | Repetitive surface protein | Likely misassembly |
