# Supplementary material for: Dominant Role of Nucleotide Substitution in the Diversification of Serotype 3 Pneumococci over Decades and during a Single Infection
Source: PLoS Genet. 2013 Oct 10;9(10):e1003868. doi: 10.1371/journal.pgen.1003868 (PMC3794909; doi:10.1371/journal.pgen.1003868)
Supplement: Table S3 — Significant differences in expression patterns between S. pneumoniae 4038 and 4039 detected using a microarray based on the S. pneumoniae TIGR4 and R6 genomes. Statistical analysis was performed using limma. The displayed p value is adjusted to reflect correction for multiple testing using the Benjamini-Hochberg method. (DOCX) [file pgen.1003868.s013.docx]

| **TIGR4 CDS** | **Gene** | **4038 Orthologue** | **Gene Product** | **4039/4038 Ratio** | ***p* Value** |
| --- | --- | --- | --- | --- | --- |
| SP_2075 | *patA* | SP4038_18230 | ABC transporter ATP-binding membrane protein | 5.09 | 8.80E-03 |
| SP_2073 | *patB* | SP4038_18210 | ABC transporter ATP-binding membrane protein | 4.79 | 8.80E-03 |
| SP_0493 | *rpoE* | SP4038_04500 | putative DNA-directed RNA polymerase, delta subunit | 0.38 | 3.85E-02 |
| SP_0492 | *-* | - | - | 0.30 | 3.54E-02 |
| SP_0373 | *-* | SP4038_03630 | putative RNA methylase family protein | 0.28 | 4.96E-02 |
| SP_0490 | *-* | SP4038_04480 | putative uncharacterized protein | 0.28 | 4.96E-02 |
| SP_0649 | *-* | - | - | 0.27 | 3.18E-02 |
| SP_0489 | *-* | SP4038_04470 | PAP2 superfamily protein | 0.27 | 2.21E-02 |
| SP_1293 | *rplS* | SP4038_11460 | 50S ribosomal protein L19 | 0.25 | 3.18E-02 |
| SP_0487 | *-* | - | - | 0.21 | 3.18E-02 |
| SP_0488 | *-* | SP4038_04460 | putative membrane protein | 0.16 | 9.94E-03 |
| SP_2214 | *tsf* | SP4038_19480 | elongation factor Ts | 0.11 | 5.44E-03 |
| SP_2215 | *rpsB* | SP4038_19490 | 30S ribosomal protein S2 | 0.08 | 5.44E-03 |

**Table S3**
