# Supplementary material for: Dominant Role of Nucleotide Substitution in the Diversification of Serotype 3 Pneumococci over Decades and during a Single Infection
Source: PLoS Genet. 2013 Oct 10;9(10):e1003868. doi: 10.1371/journal.pgen.1003868 (PMC3794909; doi:10.1371/journal.pgen.1003868)
Supplement: Table S4 — Significant differences in expression patterns between S. pneumoniae 4038 and 4039 detected using a RNA-seq. Statistical analysis was performed using DESeq. The displayed p value is adjusted to reflect correction for multiple testing using the Benjamini-Hochberg method. (DOCX) [file pgen.1003868.s014.docx]

**Table S4**

| **4038 CDS** | **Gene** | **TIGR4 Orthologue** | **Gene Product** | **4039/4038 Ratio** | ***p* Value** |
| --- | --- | --- | --- | --- | --- |
| SP4038_05850 | *-* | SP_2314 | putative uncharacterized protein | 0.07 | 9.08E-08 |
| SP4038_05840 | *-* | SP_2313 | putative uncharacterized protein | 0.08 | 1.02E-04 |
| SP4038_19490 | *rpsB* | SP_2215 | 30S ribosomal protein S2 | 0.08 | 1.15E-06 |
| SP4038_19480 | *tsf* | SP_2214 | elongation factor Ts | 0.15 | 1.83E-06 |
| SP4038_03630 | *-* | SP_0373 | putative RNA methylase family protein | 0.16 | 9.08E-08 |
| SP4038_16340 | *-* | SP_1871 | siderophore uptake ATP-binding protein | 0.17 | 2.38E-03 |
| SP4038_16330 | *-* | SP_1870 | putative iron compound ABC transporter, permease protein | 0.18 | 1.44E-03 |
| SP4038_03640 | *-* | SP_0374 | putative membrane protein | 0.18 | 1.65E-07 |
| SP4038_16350 | *-* | SP_1872 | siderophore uptake periplasmic binding protein | 0.18 | 3.83E-04 |
| SP4038_16240 | *-* | SP_1859 | nicotinamide mononucleotide transporter | 0.18 | 1.78E-02 |
| SP4038_04470 | *-* | SP_0489 | PAP2 superfamily protein | 0.20 | 1.45E-05 |
| SP4038_04460 | *-* | SP_0488 | putative membrane protein | 0.21 | 6.20E-06 |
| SP4038_04520 | *-* | SP_0496 | putative Na+/Pi-cotransporter protein | 0.21 | 2.72E-06 |
| SP4038_04680 | *dnaK* | SP_0517 | chaperone protein DnaK (heat shock protein 70) | 0.21 | 2.82E-05 |
| SP4038_04490 | *-* | SP_2283 | acetyltransferase (GNAT) family protein | 0.23 | 8.55E-03 |
| SP4038_04480 | *-* | SP_0490 | putative uncharacterized protein | 0.23 | 1.46E-03 |
| SP4038_07160 | *-* | SP_0800 | putative membrane protein | 0.24 | 4.04E-03 |
| SP4038_14150 | *rpsO* | SP_1626 | 30S ribosomal protein S15 | 0.27 | 2.38E-03 |
| SP4038_14570 | *-* | SP_1674 | putative transcription regulator | 0.29 | 1.44E-03 |
| SP4038_04690 | *-* | - | putative membrane protein | 0.29 | 2.38E-03 |
| SP4038_04500 | *rpoE* | SP_0493 | putative DNA-directed RNA polymerase, delta subunit | 0.33 | 9.98E-03 |
| SP4038_11460 | *rplS* | SP_1293 | 50S ribosomal protein L19 | 0.34 | 2.61E-02 |
| SP4038_18070 | *tgt* | SP_2058 | queuine tRNA-ribosyltransferase | 0.34 | 4.04E-03 |
| SP4038_05700 | *rplA* | SP_0631 | 50S ribosomal protein L1 | 0.34 | 2.13E-02 |
| SP4038_03420 | *clpL* | SP_0338 | putative ATP-dependent protease ATP-binding subunit ClpL | 0.35 | 1.02E-02 |
| SP4038_04670 | *grpE* | SP_0516 | GrpE protein (HSP-70 cofactor) | 0.41 | 2.13E-02 |
| SP4038_11750 | *rplJ* | SP_1355 | 50S ribosomal protein L10 | 2.27 | 1.10E-02 |
| SP4038_12590 | *guaA* | SP_1445 | GMP synthase [glutamine-hydrolyzing] | 2.37 | 2.14E-02 |
| SP4038_02480 | *infA* | SP_0232 | translation initiation factor IF-1 | 2.41 | 2.13E-02 |
| SP4038_13270 | *-* | SP_1527 | putative extracellular oligopeptide-binding protein | 2.44 | 3.31E-02 |
| SP4038_08480 | *infC* | SP_0959 | translation initiation factor IF-3 | 2.55 | 3.59E-02 |
| SP4038_04570 | *glnA* | SP_0502 | putative glutamine synthetase | 2.57 | 1.73E-02 |
| SP4038_06620 | *-* | SP_0742 | putative fatty-acid binding protein | 2.59 | 1.15E-02 |
| SP4038_12450 | *-* | SP_1428 | CHY-type zinc finger protein | 2.72 | 2.39E-02 |
| SP4038_08500 | *rplT* | SP_0961 | 50S ribosomal protein L20 | 2.81 | 9.90E-03 |
| SP4038_08510 | *gloA* | SP_0962 | putative lactoylglutathione lyase | 2.83 | 1.82E-03 |
| SP4038_02510 | *rpoA* | SP_0236 | DNA-directed RNA polymerase alpha chain | 2.86 | 4.81E-02 |
| SP4038_02520 | *rplQ* | SP_0237 | 50S ribosomal protein L17 | 2.95 | 5.28E-03 |
| SP4038_00980 | *purK* | SP_0054 | putative phosphoribosylaminoimidazole carboxylase ATPase subunit | 2.98 | 1.76E-02 |
| SP4038_02990 | *-* | SP_0287 | putative permease | 3.16 | 1.12E-02 |
| SP4038_02470 | *adk* | SP_0231 | adenylate kinase | 3.24 | 1.51E-03 |
| SP4038_18210 | *patB* | SP_2073 | ABC transporter ATP-binding membrane protein | 3.89 | 2.51E-05 |
| SP4038_18230 | *patA* | SP_2075 | ABC transporter ATP-binding membrane protein | 4.09 | 4.51E-04 |
| SP4038_19440 | *cysM* | SP_2210 | putative cysteine synthase | 4.69 | 1.46E-02 |
| SP4038_00940 | *vanZ* | SP_0049 | putative VanZ-family resistance protein | 5.44 | 8.30E-03 |
| SP4038_00890 | *purC* | SP_0044 | phosphoribosylaminoimidazole-succinocarboxamidesynthase | 6.15 | 9.37E-03 |
| SP4038_03900 | *-* | SP_0409 | putative decarboxylase | 6.17 | 8.72E-15 |
| SP4038_00900 | *-* | SP_0045 | putative phosphoribosylformylglycinamidine synthase protein | 6.42 | 3.55E-06 |
| SP4038_05570 | *-* | SP_0620 | putative extracellular solute-binding protein | 6.66 | 3.63E-04 |
| SP4038_00950 | *purH* | SP_0050 | bifunctional purine biosynthesis protein | 6.76 | 3.39E-04 |
| SP4038_03000 | *-* | SP_0288 | putative CAAX amino terminal protease family membrane protein | 7.50 | 2.38E-03 |
| SP4038_18200 | *-* | SP_2072 | putative peptidase | 7.50 | 2.81E-07 |
| SP4038_00910 | *purF* | SP_0046 | putative amidophosphoribosyltransferase precursor | 7.67 | 4.38E-07 |
| SP4038_00930 | *purN* | SP_0048 | phosphoribosylglycinamide formyltransferase | 8.68 | 9.39E-06 |
