# Supplementary material for: Dominant Role of Nucleotide Substitution in the Diversification of Serotype 3 Pneumococci over Decades and during a Single Infection
Source: PLoS Genet. 2013 Oct 10;9(10):e1003868. doi: 10.1371/journal.pgen.1003868 (PMC3794909; doi:10.1371/journal.pgen.1003868)
Supplement: Table S5 — Results of the Omnilog phenotype microarray assay. This table lists the compounds found to result in significantly higher respiration in 4039 relative to 4038. Each compound was tested at four different concentrations; that concentration at which a significant difference was observed is indicated by a number, with one being the lowest concentration and four being the highest. The displayed p value is adjusted to reflect correction for multiple testing using the Benjamini-Hochberg method. (DOCX) [file pgen.1003868.s015.docx]

**Table S5**

| **Compound** | **Concentration** | **Antimicrobial mechanism** | ***p* value** |
| --- | --- | --- | --- |
| 6-Mercaptopurine | 4 | nucleic acid analog, purine | 0.0079 |
| Acriflavine | 2 | DNA intercalator | 0.0079 |
| Boric Acid | 3 | transport, toxic anion | 0.0079 |
| Sodium Metaborate | 3 | transport, toxic anion | 0.0079 |
| 5,7-Dichloro-8-hydroxyquinoline | 1 | chelator, lipophilic | 0.0079 |
| Nordihydroguaiaretic acid | 2 | lipoxygenase, fungicide | 0.0079 |
| 3, 4-Dimethoxybenzyl alcohol | 3 | oxidizing agent, free radical-peroxidase substrate | 0.0079 |
| Aminotriazole | 3 | histidine biosynthesis, catalase | 0.0079 |
| Cefsulodin | 3 | wall, cephalosporin | 0.0079 |
| Amitriptyline | 2 | membrane, transport | 0.0079 |
| Orphenadrine | 2 | cholinergic antagonist | 0.0079 |
| D,L-Propranolol | 3 | beta-adrenergic blocker | 0.0079 |
| Proflavine | 2 | RNA synthesis | 0.0079 |
| Crystal Violet | 2 | respiration | 0.0079 |
| D,L-Methionine Hydroxamate | 3 | tRNA synthetase | 0.0113 |
| 6-Mercaptopurine | 2 | nucleic acid analog, purine | 0.0134 |
| Sodium bromate | 3 | toxic anion | 0.0134 |
| 1% sodium chloride | 1 | osmolyte | 0.0145 |
| 6-Mercaptopurine | 1 | nucleic acid analog, purine | 0.0145 |
| Domiphen bromide | 2 | membrane, detergent, cationic, fungiside | 0.0145 |
| 6-Mercaptopurine | 3 | nucleic acid analog, purine | 0.0156 |
| 2,4-Dintrophenol | 3 | respiration, ionophore, H^+^ | 0.0156 |
| 5,7-Dichloro-8-hydroxyquinoline | 2 | chelator, lipophilic | 0.0193 |
| Pentachlorophenol (PCP) | 1 | respiration, ionophore, H^+^ | 0.0237 |
| Harmane | 4 | imidazoline binding sites, agonist | 0.0242 |
