# Supplementary material for: Dominant Role of Nucleotide Substitution in the Diversification of Serotype 3 Pneumococci over Decades and during a Single Infection
Source: PLoS Genet. 2013 Oct 10;9(10):e1003868. doi: 10.1371/journal.pgen.1003868 (PMC3794909; doi:10.1371/journal.pgen.1003868)
Supplement: Table S6 — Susceptibility of isolates S. pneumoniae 99-4038, 99-4039, TIGR4 and TIGR4PUS to common antibiotics. Minimum inhibitory concentrations (MICs) were determined either used the Vitek 2 or E test methods. (DOCX) [file pgen.1003868.s016.docx]

**Table S6**

| **Antibiotic** | **Method** | **4038 MIC (mg L^-1^)** | **4039 MIC (mg L^-1^)** | **TIGR4 MIC (mg L^-1^)** | **TIGR4^PUS^ MIC (mg L^-1^)** |
| --- | --- | --- | --- | --- | --- |
| Benzylpenicillin | Vitek 2 | ≤0.06 | ≤0.06 | ≤0.06 | ≤0.06 |
| Ampicillin | Vitek 2 | ≤0.25 | ≤0.25 | ≤0.25 | ≤0.25 |
| Cefotaxime | Vitek 2 | ≤0.12 | ≤0.12 | ≤0.12 | ≤0.12 |
| Ceftriaxone | Vitek 2 | ≤0.12 | ≤0.12 | ≤0.12 | ≤0.12 |
| Levofloxacin | Vitek 2 | 0.5 | 1 | 1 | 2 |
| Erythromycin | Vitek 2 | ≤0.12 | ≤0.12 | ≤0.12 | ≤0.12 |
| Clindamycin | Vitek 2 | ≤0.25 | ≤0.25 | ≤0.25 | ≤0.25 |
| Linezolid | Vitek 2 | ≤2 | ≤2 | ≤2 | ≤2 |
| Vancomycin | Vitek 2 | 0.25 | 0.5 | 0.25 | 0.25 |
| Tetracycline | Vitek 2 | ≤0.25 | 0.5 | ≤0.25 | 0.5 |
| Cotrimoxazole | Vitek 2 | ≤10 | ≤10 | ≤10 | ≤10 |
| Ciprofloxacin | E-test | 0.75 | 2 | 1 | 4 |
