# Supplementary material for: Dominant Role of Nucleotide Substitution in the Diversification of Serotype 3 Pneumococci over Decades and during a Single Infection
Source: PLoS Genet. 2013 Oct 10;9(10):e1003868. doi: 10.1371/journal.pgen.1003868 (PMC3794909; doi:10.1371/journal.pgen.1003868)
Supplement: Table S7 — Significant differences in expression patterns between S. pneumoniae TIGR4 and the modified strain carrying the PUS (TIGR4PUS), detected using a microarray based on the S. pneumoniae TIGR4 genome. Statistical analysis was performed using limma. The displayed p value is adjusted to reflect multiple testing using the Benjamini-Hochberg method. (DOCX) [file pgen.1003868.s017.docx]

**Table S7**

| **TIGR4 CDS** | **Gene** | **4038 Orthologue** | **Gene Product** | **TIGR4^PUS^/TIGR4 Ratio** | ***p* Value** |
| --- | --- | --- | --- | --- | --- |
| SP_2073 | *patB* | SP4038_18210 | ABC transporter ATP-binding membrane protein | 2.68 | 2.25E-04 |
| SP_2075 | *patA* | SP4038_18230 | ABC transporter ATP-binding membrane protein | 2.43 | 6.07E-05 |
| SP_2074 | - | - | - | 1.61 | 6.06E-04 |
| SP_0455 | - | - | - | 1.15 | 3.10E-02 |
